# Single Cu Atom Sites on Co_3_O_4_ Activate Interfacial Oxygen for Enhanced Reactivity and Selective Gas Sensing at Low Temperature

**DOI:** 10.1002/smll.202600033

**Published:** 2026-04-13

**Authors:** Hamin Shin, Matteo D'Andria, Meng Yin, Jaehyun Ko, Ken Suzuki, Dong‐Ha Kim, Frank Krumeich, Andreas T. Güntner

**Affiliations:** ^1^ Human‐centered Sensing Laboratory Department of Mechanical and Process Engineering ETH Zürich Zürich Switzerland; ^2^ Green X‐Tech Center Green Goals Initiative Tohoku University Sendai Miyagi Japan; ^3^ Department of Materials Science and Chemical Engineering Hanyang University Ansan Republic of Korea; ^4^ Department of Chemistry and Applied Biosciences Laboratory of Inorganic Chemistry ETH Zürich Zürich Switzerland

**Keywords:** air pollutant detection, chemiresistive gas sensors, electronic materials, environmental monitoring, nanotechnology, surface engineering

## Abstract

Controlling the redox landscape of transition metal oxides is central to advancing their reactivity for heterogeneous catalysis or high‐performance gas sensing. Here, we report single Cu atom sites (1.42 wt.%) anchored on Co_3_O_4_ nanoparticles (Cu_1_‐Co_3_O_4_) that dramatically enhance reactivity and molecular sensing properties of the support at low temperature. The Cu_1_ are identified by x‐ray absorption near edge structure and feature metal–support interaction between the atomically dispersed Cu (mostly in 2+ oxidation state) and Co_3_O_4_, as revealed by x‐ray photoelectron spectroscopy. The ability of Cu_1_ to form interfacial Cu–O–Co linkages strongly reduces the temperature of lattice oxygen activation compared to CuO nanoparticles on Co_3_O_4_ (CuO_NP_‐Co_3_O_4_), as demonstrated by temperature‐programmed reduction and desorption analyses, in agreement with density functional theory calculations. To demonstrate practical impact, we deploy Cu_1_‐Co_3_O_4_ nanoparticles as a chemoresistive sensor for formaldehyde that yields more than an order of magnitude higher response than CuO_NP_‐Co_3_O_4_ and consistently outperforms state‐of‐the‐art sensors. Formaldehyde is detected down to 5 parts‐per‐billion at 50% relative humidity and 75°C with excellent selectivity over critical interferents. These results establish a strategy for activating redox‐active supports using single‐atom isolates of non‐noble nature, yielding drastically enhanced and well‐defined reactivity to promote low‐temperature oxidation reactions and selective analyte sensing.

## Graphical abstract

Atomically dispersed Cu sites on Co_3_O_4_ nanoparticles establish well‐defined Cu–O–Co interfacial linkages that promote lattice oxygen activation at markedly reduced temperatures. This structural and electronic modulation yields over twenty‐fold higher low‐temperature formaldehyde sensing responses than CuO nanoparticle analogues, enabling selective and energy‐efficient detection with earth‐abundant metals.

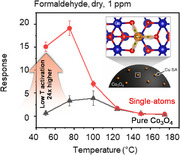

## 1 Introduction

Controlling the spatial distribution and size of active metal species on catalyst supports offers unprecedented opportunities for the tuning of surface reactivity [1, 2, 3, 4] with immediate practical impact on heterogeneous catalysis [5], energy storage [6], or molecular sensing [7, 8, 9]. In particular, down‐sizing from metal nanoparticles (NPs) to single‐atom sites (SAs) on metal oxides [10], graphene derivatives [11], or metal‐organic frameworks [12] has emerged as a transformative strategy, enabling maximal atom efficiency [13, 14], altered electronic structures [15, 16, 17], and enhanced catalytic precision [18]. These attributes are advantageous, for instance, in chemoresistive gas sensors, where SAs offer the opportunity for well‐defined surface reactivity and to explore different reaction pathways to optimize sensitivity and selectivity [19] to an analyte [20]. While noble metal SAs such as Pt [21, 22], Pd [23, 24], and Au [25, 26] have been widely explored, non‐noble transition metal SAs remain less investigated despite their abundance, thus cost‐effectiveness, and diverse redox chemistries.

Among them, Cu‐based SAs stand out as promising alternatives with proven performance for selective oxidation reactions [27, 28] and electrocatalysis, such as oxygen reduction reaction [29, 30]. Cu SAs are stabilized on supports by metal–support interactions (MSI) [27] or local coordination environments [31] that modulate their oxidation states, thereby facilitating charge transfer and catalytic activation. While these studies highlight the catalytic potential of Cu SAs, most investigations have focused primarily on reactivity trends without deeply probing how the Cu dispersion state perturbs the redox landscape of the support that is related to chemoresistive sensor signal generation. Furthermore, the role of SA‐induced interfacial chemistry has rarely been addressed beyond model oxide systems, limiting broader understanding of how atomic Cu influences host materials with active lattice oxygen.

In particular, little is known about how atomically dispersed Cu modulates the electronic structure and oxygen activation capacity of redox‐active supports compared to its nanoparticulate counterpart. The structural and electronic effects arising from different Cu configurations should be explored [32] to reveal structure–function relationships. Addressing these gaps will be critical for understanding how non‐noble metal SAs interact with support lattices and for rationalizing their broader functional implications across redox‐driven applications [33].

Here, we explore how non‐noble metal SAs fundamentally alter the redox properties of a transition metal oxide support through interfacial electronic interaction with immediate practical implications on low‐temperature heterogeneous catalysis and chemoresistive sensing of environmental pollutants. Using flame‐synthesized Co_3_O_4_ as a platform, we compare atomically dispersed Cu (Cu_1_‐Co_3_O_4_) with surface‐deposited CuO nanoparticles (Cu_NP_‐Co_3_O_4_) at identical Cu surface loading to decouple the structural and electronic consequences of atomic‐scale versus aggregated Cu species. A combination of x‐ray spectroscopy and temperature‐programmed analyses is utilized to identify the activation of lattice oxygen by Cu SAs and modifications to the Co redox landscape. To demonstrate the functional implications of our findings, we employ them exemplarily for formaldehyde gas sensing. This study bridges a mechanistic gap in non‐noble metal SA research and underscores the potential of atomic‐scale engineering in developing efficient catalysts and sustainable sensing materials.

## 2 Results

### 2.1 Stabilization of Cu Species on Co_3_O_4_: Structural Speciation

Flame‐spray pyrolysis, a combustion‐aerosol technique [34], is utilized for the fabrication of Co_3_O_4_ nanoparticles with abundant oxygen vacancies, as demonstrated for other metal oxides [35, 36], which are known to be one of the primary stabilization sites for single‐atom sites (SAs) [37, 38]. Cu SAs are decorated by wet impregnation on the surface of Co_3_O_4_ nanoparticles, where a surface loading of 1.42 wt.% Cu SAs is achieved, as determined by inductively coupled plasma mass spectrometry. This surface loading surpasses many previously reported values for Cu SAs on oxide supports prepared by conventional wet impregnation and sol‐gel methods, which often result in limited dispersion [13]. For example, Cu loading was only to 0.34 wt.% on ZSM‐5 [39] and 0.86 wt.% on CeO_2_‐TiO_2_ [27], likely due to the lower density of anchoring sites in such systems.

**FIGURE 1:**
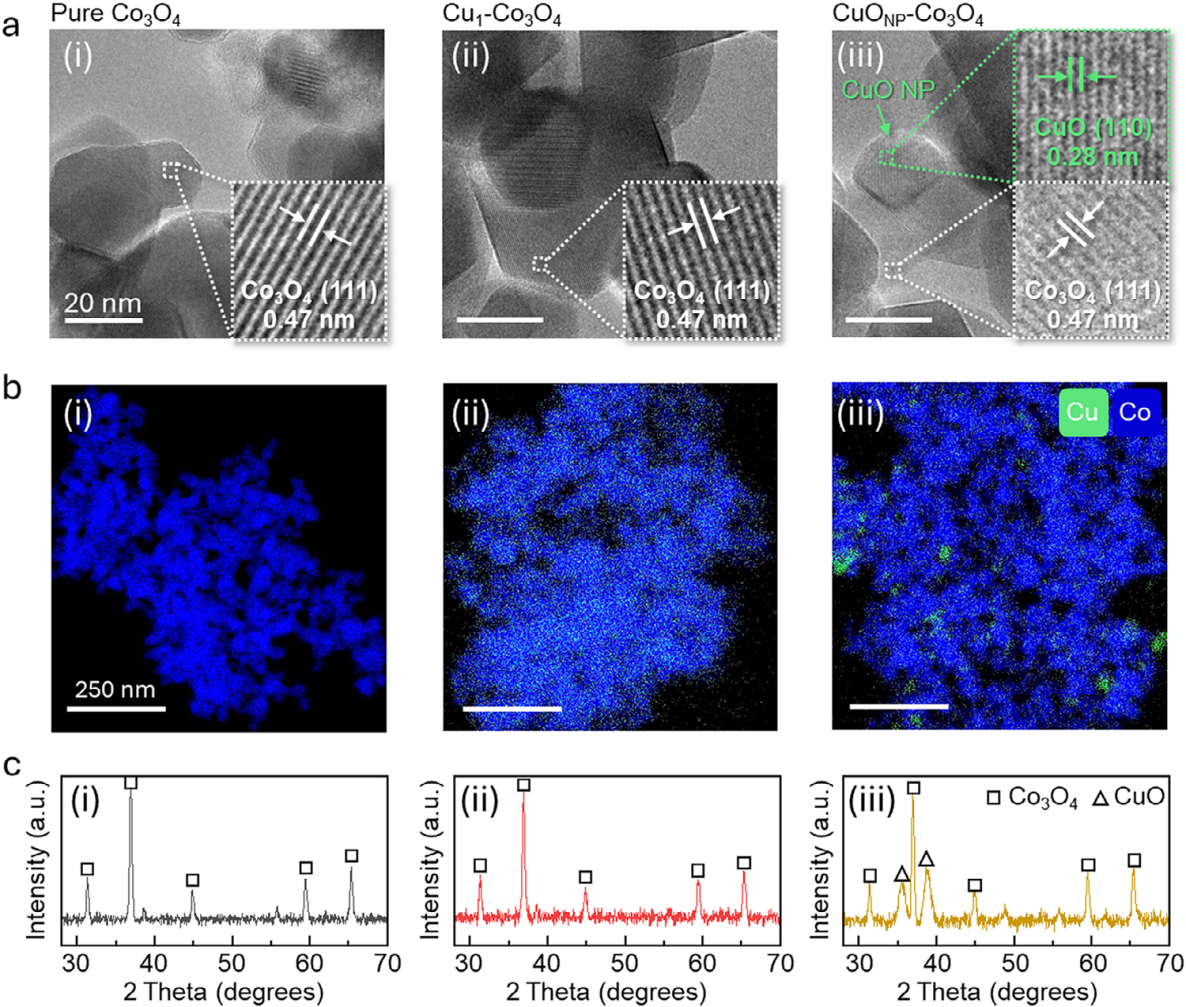
(a) High‐resolution TEM images with magnifications and labelled lattice fringes in the insets of (i) pristine Co_3_O_4_, (ii) Cu_1_‐Co_3_O_4_, and (iii) CuO_NP_‐Co_3_O_4_. (b) STEM‐EDS elemental mapping images indicating Co (blue) and Cu (green). (c) Powder XRD patterns.

A high‐resolution transmission electron microscopy image (Figure 1a(i)) of the pure Co_3_O_4_ reveals well‐defined crystalline nanoparticles with faceted shape and measured lattice spacing of 0.47 nm (see inset), corresponding to the (111) planes of spinel Co_3_O_4_ (JCPDS No. 42–1467). After the introduction of Cu species (Cu_1_‐Co_3_O_4_, Figure 1a(ii)), the overall morphology of the Co_3_O_4_ nanoparticles and their crystal lattice remain unchanged. This suggests the successful anchoring of Cu species on the Co_3_O_4_ support, forming a Cu_1_‐Co_3_O_4_ heterostructure. For comparison, we also examined CuO NP‐loaded samples (CuO_NP_‐Co_3_O_4_, Figure 1a(iii)), where particles with a lattice spacing of 0.28 nm are observed, which can be attributed to the (110) plane of CuO (JCPDS No. 48–1548).

Elemental mapping (EDS) further corroborates the presence of well‐dispersed Cu species anchored on the Co_3_O_4_ lattice. Compared to the pure Co_3_O_4_ sample (Figure 1b(i)), the Cu_1_‐Co_3_O_4_ (Figure 1b(ii); Figure S1) displays a homogeneous dispersion of Cu (green). The corresponding EDS spectra (Figure S2) confirm the presence of Cu alongside Co. In contrast, CuO_NP_‐Co_3_O_4_ (Figure 1b(iii)) features localized Cu‐rich regions that indicates the presence of clusters or NPs attached to the Co_3_O_4_, despite identical Cu surface loading, as also confirmed by EDS spectra (Figure S2 vs. Figure S3)

Powder x‐ray diffraction (XRD) patterns (Figure 1c) are collected to analyze the crystal structure and phase composition of the pristine and Cu‐loaded Co_3_O_4_ samples. All three samples show diffraction peaks that are associated with the spinel Co_3_O_4_ phase (squares), indicating that the crystal structure of the support remains preserved upon Cu addition. The diffraction peaks of Cu_1_‐Co_3_O_4_ (Figure 1c(ii)) closely resemble those of the pristine Co_3_O_4_ (Figure 1c(i)), suggesting that the Cu species in Cu_1_‐Co_3_O_4_ do not alter the crystalline bulk structure. Note that no peak shifts were identified (Figure S4), supporting the exclusive presence of Cu on the surface of Co_3_O_4_. In contrast, the CuO_NP_‐Co_3_O_4_ sample (Figure 1c(iii)) shows additional reflections (triangles) at 2θ = 35.5° and 39.0° that can be indexed to the ($\bar{1}$11) and (111) planes of monoclinic CuO, respectively, indicating the presence of crystalline CuO NPs anchored (Figure 1b(iii)) on the Co_3_O_4_ support.

To quantitatively assess the structural evolution of Co_3_O_4_ upon Cu impregnation, the Co_3_O_4_ crystal sizes of the samples are estimated from Figure 1c using the Scherrer equation. As shown in Figure S5, pristine Co_3_O_4_ exhibits the largest average crystallite size of 25.3 nm, in fair agreement with literature [40]. In the case of Cu_1_‐Co_3_O_4_, the crystallite size decreases slightly to 23.1 nm, suggesting that the Cu SAs act as defects, thus interfering crystal growth during annealing (see Experimental section) possibly by stabilizing high‐energy surfaces or introducing local strain [41]. In the CuO_NP_‐Co_3_O_4_ sample, the crystallite size further decreases to 21.3 nm, implying that surface‐anchored CuO NPs (Figure 1b(iii)) limit the coalescence and growth of Co_3_O_4_ crystallites possibly by acting as a physical barrier [21, 42]. This trend is in line with prior observations on CuO_x_ clusters on Co_3_O_4_ [40] and TiO_x_ [43] or Y_x_O_y_ [44] on ZnO.

### 2.2 Atomic Dispersion and Local Coordination of Cu Sites in Cu_1_‐Co_3_O_4_

**FIGURE 2:**
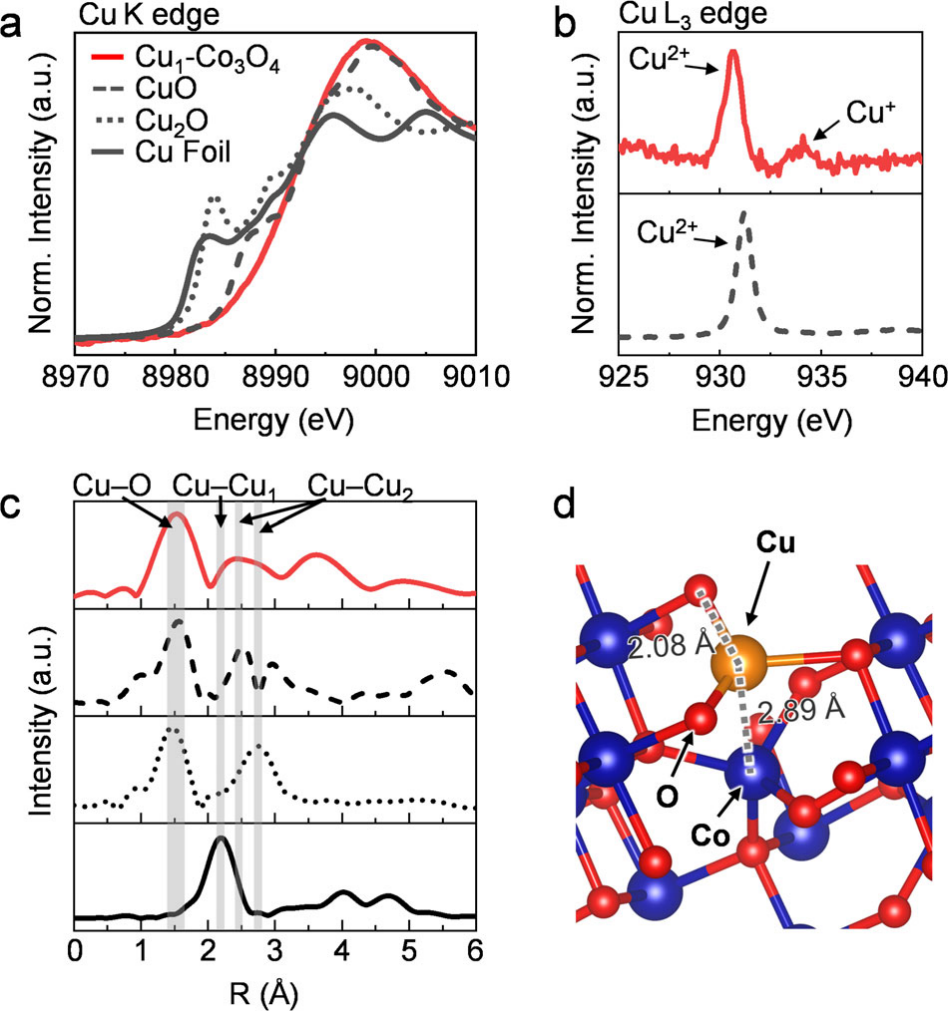
(a) XANES spectra of Cu_1_‐Co_3_O_4_ and reference powder/foil samples. (b) NEXAFS spectrum of Cu_1_‐Co_3_O_4_ and reference CuO powder at the Cu L_3_‐edge under ultra‐high vacuum. (c) Fourier‐transformed Cu K‐edge EXAFS spectra of Cu_1_‐Co_3_O_4_ and various reference samples. Displayed by gray‐shaded areas are the positions of Cu–O [53], first shell Cu–Cu and second shell Cu–Cu [31]. (d) Atomic model of Cu SA (orange) anchored on Co_3_O_4_ surface, showing average Cu─O and Cu─Co bond distances of 2.08 and 2.89 Å, respectively, as indicated by dotted lines. Illustration shows exemplary Cu‐O_3_ coordination, where Cu‐O_4_ coordination is also possible, according to Table S1.

To elucidate the oxidation state and local coordination environment of Cu species anchored on Co_3_O_4_, x‐ray absorption near‐edge structure (XANES) and extended x‐ray absorption fine structure (EXAFS) spectroscopy are performed at the Cu K‐edge, along with near‐edge x‐ray absorption fine structure (NEXAFS) spectroscopy at the Cu L_3_‐edge (red lines, Figure 2). For comparison, CuO (black dashed line), Cu_2_O (dotted), and Cu foil (solid) are measured as reference samples, with their specifics being reported in the Experimental section.

As shown in the XANES spectra (Figure 2a, magnified in Figure S6), the white line position of Cu_1_‐Co_3_O_4_ lies between those of Cu_2_O and CuO, but more closely resembles the edge of CuO. This suggests that the Cu species predominantly exist in the Cu^2+^ oxidation state. In fact, the average oxidation state of Cu in Cu_1_‐Co_3_O_4_ is 1.69, obtained by linear combination fitting of the XANES spectra (Figure S7) [17]. The result is supported by the Cu 2p x‐ray photoelectron spectrum of Cu_1_‐Co_3_O_4_ and CuO_NP_‐Co_3_O_4_ in Figure S8. Notably, no discernible shoulder or pre‐edge feature associated with metallic Cu^0^ is observed, excluding the presence of Cu clusters or NPs [31, 45]. These results indicate that Cu is uniformly oxidized and does not form metallic aggregates on the Co_3_O_4_ surface. It is also worth noting that CuO and Cu_2_O display a distinct white line at the Cu K‐edge, arising from dipole‐allowed 1s → 4p transitions, as well as weak pre‐edge shoulders arising from 3d–4p hybridization in distorted coordination environments [46]. In contrast, such spectral features are not observed for Cu_1–_Co_3_O_4_, suggesting a more centrosymmetric oxygen coordination geometry around Cu, limiting 3d–4p mixing and thereby reducing white line asymmetry [47]. Such local structure is consistent with the presence of atomically dispersed Cu^2+^ centers on the Co_3_O_4_ support.

The NEXAFS spectrum (Figure 2b) further confirms the oxidized state of Cu in Cu_1_‐Co_3_O_4_. A pronounced white line feature at around 931 eV is observed at the Cu L_3_‐edge, attributed to the dipole‐allowed 2p_3/2_ → 3d electronic transitions in Cu^2+^ [48]. The intensity and energy position of this feature are closely aligned with those of the CuO reference spectrum, indicating that the Cu species in Cu_1_‐Co_3_O_4_ adopt a similar oxidation state. While the L_3_‐edge peak maximum of Cu_1_‐Co_3_O_4_ occurs at reduced energy compared to CuO, peak position alone is insufficient to ascribe a change in formal oxidation state [49]. However, a weak shoulder is discernible near ∼934 eV, which we assign to the presence of Cu^+^ species in agreement with the first‐derivative K‐edge analysis (Figure S7), confirming the co‐existence of slightly reduced Cu species in Cu_1_‐Co_3_O_4_ [50, 51]. The white line in Cu_1_‐Co_3_O_4_ appears slightly sharper and more symmetric compared to that of bulk CuO, suggesting that the Cu species experience a more homogeneous and possibly more symmetric ligand field [52]. This distinction implies that the Cu^2+^ centers in Cu_1_‐Co_3_O_4_ are not part of a bulk‐like CuO phase but are rather atomically dispersed, in agreement with Figure 1, and coordinated to surface oxygen atoms in the Co_3_O_4_ matrix [47].

To probe the local coordination structure, Fourier‐transformed (FT)‐EXAFS analysis is carried out (Figure 2c). The FT‐EXAFS spectrum of Cu_1_‐Co_3_O_4_ (red line) shows a dominant peak at approximately 1.5 Å, corresponding to the Cu–O first coordination shell [53]. Importantly, no distinct peaks are observed beyond this region, especially not at ∼2.2 Å as with Cu foil (black solid line), that would indicate Cu–Cu scattering [31]. This absence of Cu–Cu scattering provides further evidence that the Cu species are atomically dispersed and not aggregated as NPs or clusters, as similarly identified for Cu SAs on TiO_2_ [54] or Ni on SiO_2._ [55] In fact, CuO (black dashed line) and Cu_2_O (black dotted line) exhibit distinct second‐shell features above 2 Å due to Cu–Cu coordination in their extended lattice structures.

To gain further structural insight, quantitative EXAFS curve fitting is performed in R‐space using a two‐shell model comprising Cu–O and Cu–Co scattering paths, based on the Co_3_O_4_ crystal framework (Figure S9). The fitting results show that the experimental data were well reproduced with this model, further supporting the local coordination of Cu^2+^‐dominant species within the oxide matrix. The fitted parameters are summarized in Table S1. The coordination number of Cu–O is found to be approximately 3.5, suggesting a planar geometry. The fitted bond distance for Cu–O is 2.08 Å, consistent with literature values for Cu^2^^+^─O bonds in oxide environments [53]. Importantly, the fit requires a Cu–Co scattering path with a coordination number 1 at 2.89 Å, which accounts for the phase‐uncorrected feature at ∼2.4 Å in the Fourier‐transformed spectrum (Figure 2c). This assignment excludes Cu–Cu contributions and uniquely points to interfacial Cu–O–Co linkages, in agreement with literature [54].

Collectively, these results strongly indicate that Cu species in Cu_1_‐Co_3_O_4_ are present as atomically dispersed Cu^2+^ centers, coordinated exclusively to oxygen atoms, with no detectable formation of metallic or oxide‐based Cu aggregates. This local coordination environment is illustrated in Figure 2d (alternative view in Figure S10), showing a Cu^2+^ center stabilized by oxygen ligands at the Co_3_O_4_ (001) facet, consistent with the configuration suggested by the EXAFS fitting. The atomically dispersed Cu^2+^ sites stabilized on the flame‐made Co_3_O_4_ support are expected to serve as unique active centers for catalytic and gas‐sensing applications, as explored below.

### 2.3 Surface Redox Properties and Metal–Support Interaction

**FIGURE 3:**
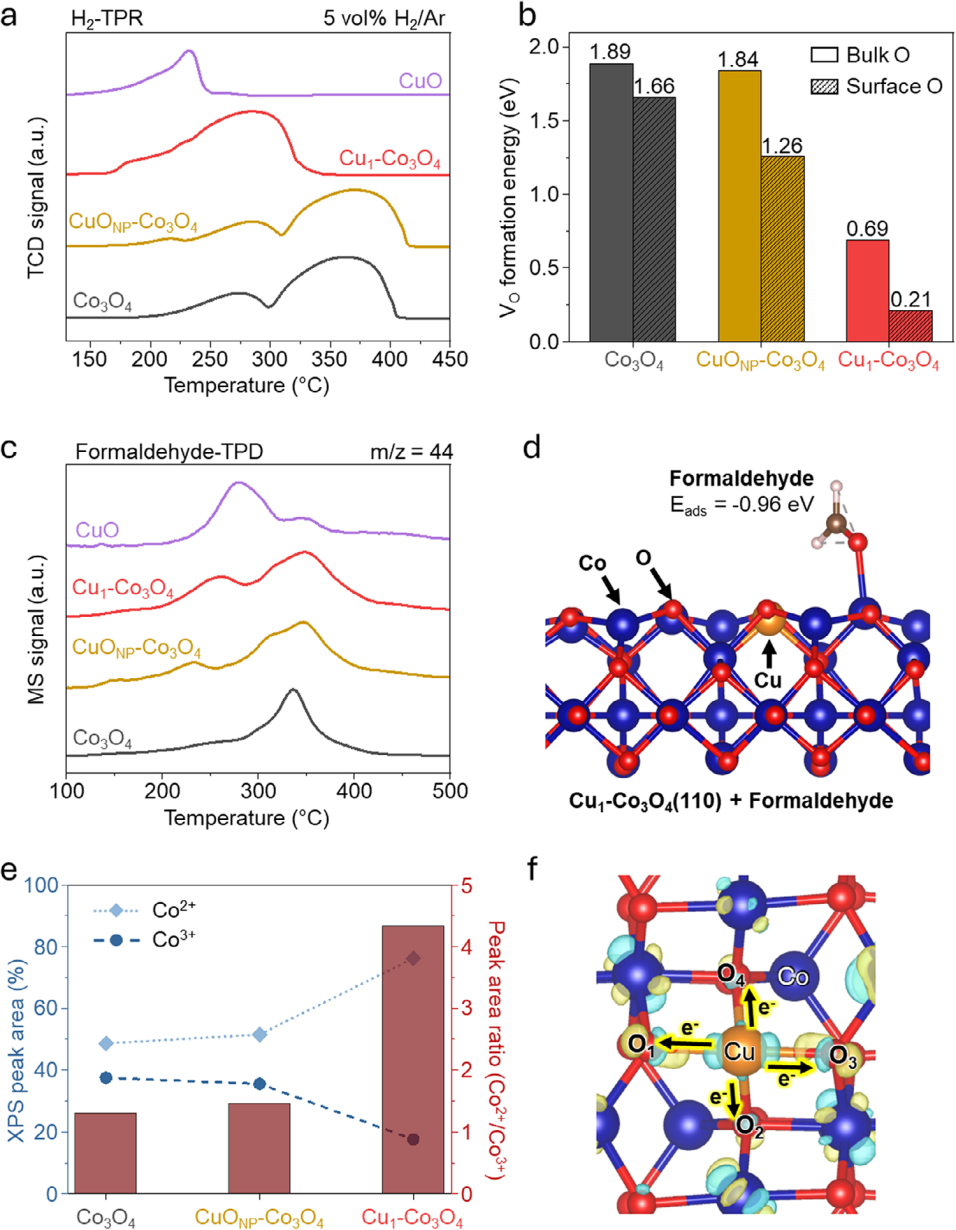
(a) H_2_‐TPR profiles of pure Co_3_O_4_, CuO_NP_‐Co_3_O_4_, Cu_1_‐Co_3_O_4_ and CuO under 5 vol.% H_2_/Ar. (b) Corresponding DFT‐calculated oxygen vacancy formation energies for bulk and surface oxygen sites. (c) Formaldehyde‐TPD profiles (m/z = 44, CO_2_) of pure Co_3_O_4_, CuO_NP_‐Co_3_O_4_, Cu_1_‐Co_3_O_4,_ and CuO under 10 ppm formaldehyde in dry air. (d) Optimized formaldehyde adsorption configuration on the Cu_1_‐Co_3_O_4_ surface. (e) Peak areas of Co^3+^ and Co^2+^ (symbols, left ordinate), and their ratios (bars, right ordinate) in pure Co_3_O_4_, CuO_NP_‐Co_3_O_4,_ and Cu_1_‐Co_3_O_4_ determined from XPS analysis (Figure S14). (f) Charge density difference of Cu_1_‐Co_3_O_4_. Blue regions indicate electron depletion, and yellow regions indicate the electron accumulations, and the isosurface is set to 0.02 eÅ^−3^.

To explore how the dispersion state of Cu modulates the surface reactivity and the degree of MSI, H_2_‐temperature‐programmed reduction (H_2_‐TPR), formaldehyde‐temperature‐programmed desorption (formaldehyde‐TPD), and x‐ray photoelectron spectroscopy (XPS) analyses are conducted (Figure 3).

The H_2_‐TPR results (Figure 3a) reveal differences in reduction behaviors depending on the Cu configuration by probing the temperature at which the Co_3_O_4_ surface and near‐surface oxygen species become reducible in H_2_, thus providing a comparative measure of low‐temperature surface redox accessibility (reducibility) across Cu_1_‐Co_3_O_4_ and CuO_NP_‐Co_3_O_4_. Both pristine Co_3_O_4_ (black) and CuO_NP_‐Co_3_O_4_ (yellow) display nearly identical two‐step reduction profiles, corresponding to the sequential reduction of Co^3+^ to Co^2+^ (∼275°C) and Co^2+^ to Co^0^ (∼360°C) [56]. In CuO_NP_‐Co_3_O_4_, an additional minor reduction peak near 210°C can be attributed to the surface reduction of CuO NPs. This assignment is consistent with the H_2_‐TPR profile of bulk CuO (purple), which exhibits a two‐step reduction starting at 170°C with a peak at 210°C that corresponds to the Cu^2+^ → Cu^+^ and Cu^+^ → Cu^0^ transitions, respectively [57]. However, the stronger similarity with pristine Co_3_O_4_ implies that the presence of CuO NPs hardly affects the reducibility of the Co_3_O_4_ matrix. In stark contrast, Cu_1_‐Co_3_O_4_ (red) exhibits a single reduction band with an onset at ∼170 and a peak at ∼275°C, significantly earlier than in the other Co_3_O_4_‐based samples and more pronounced than CuO. The low‐temperature position of this band suggests the sequential reduction of Co^δ+^ at lower temperatures due to the presence of atomically dispersed Cu. This is reminiscent of the temperature shift observed in Co_3_O_4_ loaded with amorphous or sub‐nanometer CuO_x_ clusters, where a distinct reduction peak appeared at 160°C, but only at ≥3 wt.% Cu loading [40].

We suggest that the incorporation of atomically dispersed Cu in the Co_3_O_4_ matrix increases the mobility of surface lattice oxygen by modulating the local electronic structure and promoting the formation of oxygen vacancies, thereby facilitating redox processes at lower temperatures [58, 59]. This trend is also evident in the O 1s XPS spectra (Figure S11), where the O_chem_ to lattice oxygen peak area ratio increases from 0.48 in CuO_NP_‐Co_3_O_4_ to 0.85 in Cu_1_‐Co_3_O_4_. O_chem_ denotes the higher binding energy O 1s contribution assigned to oxygen‐containing surface species [60], including chemisorbed oxygen and hydroxyl species, that can interact with such oxygen vacancies. This enhancement may reflect a larger contribution from such surface species in Cu_1_‐Co_3_O_4_, which can arise from a more strongly perturbed Cu–O–Co local environment and oxygen‐deficient surface motifs that facilitate hydroxyl formation [61, 62], in line with the earlier low‐temperature reducibility observed in H_2_‐TPR. Density functional theory (DFT) calculations confirm that atomically dispersed Cu activates interfacial oxygen removal from the Co_3_O_4_ lattice (Figure 3b; Figure S12). In pure Co_3_O_4_, the oxygen vacancy formation energy is calculated to be 1.89 eV for bulk O and 1.66 eV for surface O. Also, CuO_NP_‐Co_3_O_4_ shows only a modest decrease to 1.84 and 1.26 eV, indicating limited perturbation of the host lattice by CuO NPs, in agreement with H_2_‐TPR in Figure 3a. In contrast, Cu_1_‐Co_3_O_4_ exhibits a pronounced reduction to 0.69 eV for bulk O and 0.21 eV for surface O. The much lower vacancy formation energies indicate that the Cu–O–Co interfacial motif substantially destabilizes lattice oxygen, with its strongest effect at the surface.

To evaluate how these structural changes influence surface adsorption properties, formaldehyde‐TPD measurements are performed (Figure 3c). Interestingly, both Cu_1_‐Co_3_O_4_ and CuO_NP_‐Co_3_O_4_ exhibit enhanced CO_2_ evolution (as a product of formaldehyde oxidation) compared to pristine Co_3_O_4_, including higher desorption intensities and the appearance of low‐temperature desorption features (∼230°C). Pure CuO also shows significant CO_2_ release, particularly at lower temperatures, indicating that the enhanced formaldehyde reactivity in CuO_NP_‐Co_3_O_4_ can be largely attributed to the intrinsic surface reactivity of CuO itself. These observations suggest that both types of Cu species, atomically dispersed and nanoparticulate, contribute similarly to surface adsorption and activation of formaldehyde. Yet, the low‐temperature desorption feature appears more pronounced for Cu_1_‐Co_3_O_4_, suggesting that Cu SAs provide a more reactive adsorption environment.

DFT calculations support this interpretation (Figure 3d; Figure S13). On pure Co_3_O_4_, formaldehyde adsorption gives an E_ad_ of −0.86 eV, whereas in Cu_1_‐Co_3_O_4_ the most stable configuration is found at a Co site adjacent to Cu, with a stronger E_ad_ of −0.96 eV. This result indicates that the Cu–O–Co environment strengthens formaldehyde binding not by adsorption directly on Cu, but by creating a more reactive neighboring Co site. In the CuO_NP_‐Co_3_O_4_ model, the preferred adsorption site is situated at the edge of the CuO‐Co_3_O_4_ boundary, although the calculated adsorption energy (−0.86 eV) remains weaker than in Cu_1_‐Co_3_O_4_. A complete screening of adsorption configurations and energies for all models is provided in Figure S13. Note that our model for CuO_NP_‐Co_3_O_4_ only considered small CuO domains, while the real sample also contains some larger CuO domains (Figure 1).

To directly probe electronic interactions between Cu and the lattice‐based Co ions, XPS analysis of the Co 2p region was conducted (Figure S14). The deconvolution results in Figure 3e reveal that the Co^2+^/Co^3+^ ratio remains largely unchanged in CuO_NP_‐Co_3_O_4_ compared to pristine Co_3_O_4_. In contrast, Cu_1_‐Co_3_O_4_ exhibits a significantly higher Co^2+^ fraction, indicating a partial reduction of Co^3+^. This shift suggests electron transfer from Cu^2+^ to Co^3+^ in the Co_3_O_4_ lattice through interfacial oxygen, as suggested also by Cu 2p XPS spectrum (Figure S8a), facilitated by the intimate atomic‐level contact and interfacial Cu–O–Co electronic coupling. These findings support that Cu SAs induce enhanced MSI compared to nanoparticulate CuO NPs, as also confirmed by charge density difference analysis in Figure 3f: In Cu_1_‐Co_3_O_4_, each Cu^2+^ is stabilized in direct contact with the Co_3_O_4_ surface, maximizing the interfacial area and enabling efficient and direct charge transfer from Cu^2+^ to the coordinating interfacial oxygen of Co_3_O_4_ lattice. Bader charge analysis of the Cu SA site further indicates clear electron depletion, with charge redistributed to the surrounding oxygen atoms (Table S2). In CuO_NP_‐Co_3_O_4_, CuO NPs, only interface‐near Cu ions interact with the support, resulting in weaker MSI and negligible influence on the Co_3_O_4_ surface electronic structure. This agrees with the earlier and more consolidated reduction peak in the H_2_‐TPR profile of Cu_1_‐Co_3_O_4_ (Figure 3a), caused by formation of labile oxygen intermediates at the electron‐rich Cu–O–Co interface. This ability to modulate the redox behavior of the oxide support at such Cu SA loading (i.e., 1.42 wt.%) is particularly relevant for catalysis, as MSI has been shown to yield exceptional activity in Cu SA systems for oxidation reactions [27] as well as in noble metal SAs under similar interfacial conditions [63, 64].

### 2.4 Chemoresistive Formaldehyde Sensing Performance

To evaluate the practical implications of Cu_1_‐Co_3_O_4_ with its enhanced reactivity for molecular sensing, we investigate its performance toward low‐temperature detection of redox‐active gas species. Formaldehyde is selected as a probing molecule due to its carcinogenic nature [65] and relevance as an air pollutant. Therefore, Cu_1_‐Co_3_O_4_ NPs are deposited by spin coating onto an alumina substrate with interdigitated Pt electrodes.

**FIGURE 4:**
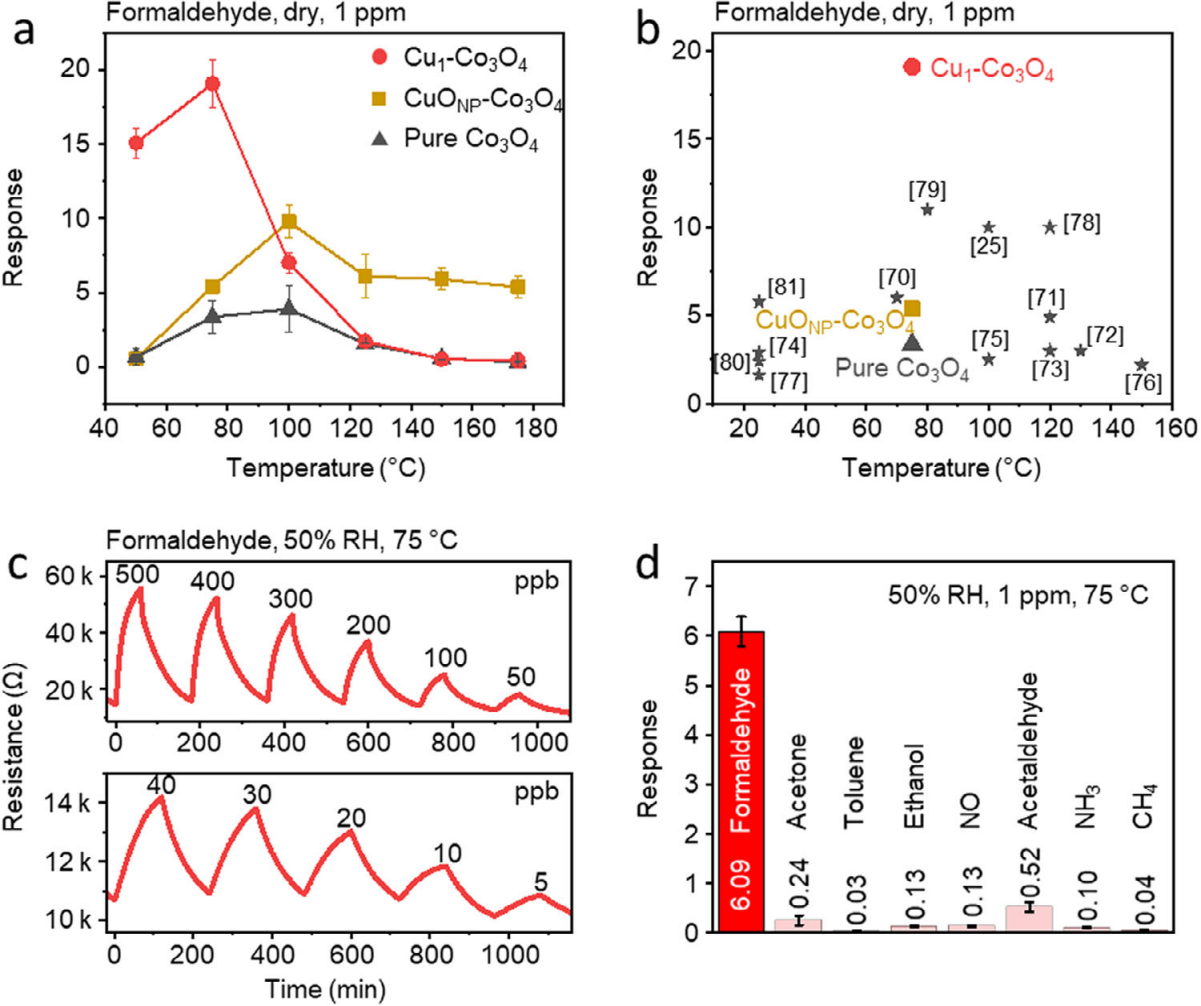
(a) Temperature‐dependent response of Cu_1_‐Co_3_O_4_ and reference samples toward 1 ppm formaldehyde under dry air condition between 50°C and 170°C. (b) Comparison of the formaldehyde sensing responses (at 1 ppm) of Cu_1–_Co_3_O_4_ and representative formaldehyde sensors reported in the literature that operate below 150°C [25, 70, 71, 72, 73, 74, 75, 76, 77, 78, 79, 80, 81]. (c) Dynamic resistance profiles of Cu_1_‐Co_3_O_4_ sensor upon exposure to varying concentrations of formaldehyde (5 to 500 ppb) at 50% RH and 75°C. (d) Cu_1_‐Co_3_O_4_ sensor response to formaldehyde and various common indoor air interferents under 50% RH and 1 ppm. Symbols and error bars shown in (a) and (d) represent the averages and standard deviations from three independently fabricated sensors, respectively.

The temperature‐dependent sensor response of Cu_1_‐Co_3_O_4_ (red circles), CuO_NP_‐Co_3_O_4_ (yellow squares), and Co_3_O_4_ (black triangles) to 1 ppm of formaldehyde in dry air is shown in Figure 4a. Most importantly, Cu_1_‐Co_3_O_4_ features a high response of 15.1 already at 50°C, that is more than an order of magnitude higher than CuO_NP_‐Co_3_O_4_ (0.5) and Co_3_O_4_ (0.6). This high response at reglatively low temperature is particularly striking and aligns well with the enhanced activation of lattice oxygen (Figure 3a) and MSI (Figure 3b,c) associated with the Cu SAs. These changes seem to collectively lower the activation energy barrier for formaldehyde oxidation, allowing an efficient gas–solid interaction at reduced temperature. In fact, the apparent activation energy for formaldehyde oxidation on Cu_1_‐Co_3_O_4_ (45.5 kJ mol^−1^) is significantly lower compared to CuO_NP_‐Co_3_O_4_ (108 kJ mol^−1^), as evidenced by kinetic analysis (Figure S15). In contrast, CuO_NP_‐Co_3_O_4_ features weaker MSI, as evidenced by the nearly unchanged Co oxidation state (Figure 3c) and TPR profile, similar to Co_3_O_4_. As a result, the optimal sensing temperature of CuO_NP_‐Co_3_O_4_ remains higher, and the sensor response to formaldehyde is generally lower compared to Cu_1_‐Co_3_O_4_ (Figure 4a). Note that the optimal sensing response occurs at much lower temperatures than formaldehyde‐TPD (Figure 3b), which is measured under oxygen‐free helium with pre‐adsorbed formaldehyde (see Experimental).

Lower operational temperature has practical implications, as it allows for portable or battery‐powered gas sensing devices [66], as needed for instance in medical diagnosis [67], where minimizing power consumption directly translates to longer device lifetime and broader deployment flexibility. For practical implementation beyond the sensing material and operation temperature, key considerations include the heater and actuator power budget, compact packaging with sampling and flow control, robust on‐board electronics with wireless control and data logging, and compensation for humidity and ambient temperature. Such aspects have been thoroughly considered in a previous study with a handheld analyzer, indicating that these system‐level requirements are achievable in portable formats [66]. In addition, robustness to environmental fluctuations is critical as temperature (Figure 4a) affects responsiveness. For practical deployment, our Cu_1_‐Co_3_O_4_ NPs may be combined with microhotplates and active temperature control to minimize the effect of environmental fluctuations [68], shorten response time, and reduce power consumption [69].

Remarkably, the Cu_1_‐Co_3_O_4_ features a maximum response of 19.1 at 75°C, with high reproducibility of ±8.3% standard deviation in three independently produced sensors. This is consistently higher than other formaldehyde sensors [25, 70, 71, 72, 73, 74, 75, 76, 77, 78, 79, 80, 81] operated at similarly low temperature (e.g., ≤150°C), as shown in Figure 4b and Table S3, emphasizing the excellent sensing properties of Cu SAs on Co_3_O_4_. For instance, a MnO_2_‐modified SnO_2_ sensor exhibited a response of approximately 12 to 1 ppm formaldehyde at 80°C [79], and another study demonstrated a porous 3D ZnO structure achieving room‐temperature detection of formaldehyde under visible light with a response of 5.6 to 1 ppm [77]. Note that higher response values have been reported only for sensors at higher temperatures (≥250°C) [21, 82]. The response values of Cu_1_‐Co_3_O_4_ toward formaldehyde may be further increased by careful optimization of surface Cu_1_ loading, as demonstrated in previous studies [21, 40].

To further challenge our Cu_1_‐Co_3_O_4_ sensor under more realistic gas environments with relative humidity (RH), we show in Figure 4c the sensor resistance change at 75°C upon consecutive formaldehyde exposure to concentrations between 500 and 5 parts‐per‐billion (ppb) at 50% RH. The sensor exhibits a well‐defined, concentration‐dependent response. Even at 5 ppb, that covers the strictest regulatory limits (i.e., 8 ppb in France) [83], a clearly distinguishable response (7.1% with a signal‐to‐noise ratio of ∼300) was obtained reproducibly (Figure S16). Note that the theoretical detection limit is calculated to be 50 ppt (see Experimental). Importantly, the resistance baseline is always recovered, indicating a fully reversible surface‐analyte interaction. The sensor shows a response time of ∼36 min and a recovery time of ∼76 min. These response and recovery times are sufficient for scheduled monitoring of indoor formaldehyde in environments where concentration changes occur over several hours, such as refurbished buildings, clinical facilities, or furniture workshops [84]. For instance, in a residential field study in the United States [85], the formaldehyde level increased gradually after ventilation was stopped, taking about 5.5 h to stabilize at 56 µg m^−3^ (∼46 ppb), whereas with continuous ventilation the concentration remained at 38 µg m^−3^ (∼31 ppb). These observations emphasize that the relatively slow response and recovery dynamics of the Cu_1_‐Co_3_O_4_ sensor align with the characteristic time scale of indoor formaldehyde fluctuations, where occupational safety regulations typically define exposure limits based on 8‐h averages (750 ppb for Occupational Safety and Health Administration [86] or 300 ppb in European Union [87]), thus fulfilling the requirements for continuous, low‐power environmental monitoring rather than instantaneous leak detection.

We also tested the sensor with eleven consecutive cycles of 100 ppb of formaldehyde at 50% RH over 2500 min (i.e., almost 2 days, Figure S17), and the sensor showed repeatable responses without any performance degradation. The same was observed over an 8‐day continuous measurement of 1 ppm formaldehyde at 50% RH (Figure S18) where the response was 5.85 ± 0.16 (average ± standard deviation) without any systematic trend that would have indicated deterioration. Humidity effect was evaluated at 0%, 50%, and 90% RH, yielding responses of 19.1, 6.1, and 2.2, respectively (Figure S19). The decrease at higher humidity is consistent with previous reports on Co_3_O_4_ sensors [40], yet the sensor retains measurable response even at ppb‐level concentrations (Figure 4c).

Selectivity is a critical parameter for indoor air sensing, where formaldehyde must be reliably distinguished from a wide range of volatile organic and inorganic compounds, such as ethanol, acetone, toluene, ammonia, and nitrogen monoxide. These compounds are usually found in household, office, or industrial environments at similar or higher concentrations than formaldehyde. Many of these interferents, especially small alcohols or ketones, exhibit overlapping oxidation reactivity on metal oxide surfaces, often resulting in cross‐sensitivity [82] and false positives. To address this, we evaluate the absolute sensor response of Cu_1_‐Co_3_O_4_ to several representative indoor interferents at 75°C and 1 ppm under 50% RH (Figure 4d). Among all tested species, the response to formaldehyde is, at least, an order of magnitude and up to 140 times higher (Table S4). This pronounced selectivity could be attributed to the intrinsically lower activation energy of formaldehyde oxidation on Cu–Co oxide interfaces, which promotes its preferential reaction over other competing analytes such as other aldehydes, alcohols, and ketones [40]. This highlights the intrinsic chemical selectivity of the sensor toward formaldehyde under humid conditions. These results support the potential of chemoresistive sensors based on single‐atom isolates with well‐defined reactivity for selective analyte detection at low operational temperature. Note that selectivity may be further enhanced with catalytic [88] or sorption‐based [89] filtering systems, if needed, to minimize background interference in real‐world environments.

## 3 Conclusion

This work demonstrates that atomic dispersion of non‐noble metals can substantially enhance redox activity in oxide supports, traditionally associated with noble metal systems. By anchoring single Cu atom sites on Co_3_O_4_ we uncover a clear shift in lattice oxygen activity and cobalt oxidation states, arising from interfacial Cu–O–Co coupling, compared to analogues of Co_3_O_4_ loaded with CuO nanoparticles. Spectroscopic and temperature‐programmed analyses reveal that Cu_1_‐Co_3_O_4_ exhibits enhanced reducibility and oxygen activation at markedly lower temperatures over CuO_NP_‐Co_3_O_4_ and Co_3_O_4_, consistent with electronic perturbation of the support, as confirmed by DFT calculations. These redox shifts are reflected in functional performance: Cu_1_‐Co_3_O_4_ sensors show an order of magnitude higher formaldehyde response at temperatures as low as 50°C, and consistently outperform other state‐of‐the‐art sensors. Importantly, the well‐defined reactivity of the Cu SA yields high formaldehyde selectivity over other molecule classes, including alcohols, ketones, aromatic compounds, and aldehydes. Together, these findings establish that atomic‐scale engineering not only enables fundamental control over support chemistry, but also elevates functional outcomes. We foresee that operando x‐ray spectroscopy could directly link this redox tuning concept to measurable sensor performance under sensing‐relevant conditions, enabling a more predictive design rule for earth‐abundant single‐atom sites on oxide supports.

## 4 Experimental Section

### 4.1 Synthesis of Pure Co_3_O_4_

Pure Co_3_O_4_ nanoparticles were synthesized using a flame‐spray pyrolysis (FSP) reactor that has been described elsewhere [90]. Briefly, a precursor solution of 0.2 m cobalt(II) 2‐ethylhexanoate (65 wt.% in mineral spirits, Sigma–Aldrich, Switzerland) dissolved in xylene (isomeric mixture, VWR Chemicals, Switzerland) was introduced through a capillary at a feed rate of 5 mL min^−1^ [40]. The solution was atomized by an oxygen dispersion flow of 5 L min^−1^, with a nozzle pressure drop of 1.6 bar, generating a fine spray. This spray underwent oxidation with the help of a flamelet sustained by CH_4_ (1.25 L min^−1^, Methane 2.5, PanGas, Switzerland) and O_2_ (3.25 L min^−^^1^, PanGas, Switzerland), while an additional O_2_ sheath flow (5 L min^−1^) provided flame stabilization. The resulting nanoparticles were collected on a water‐cooled glass fiber filter (257 mm diameter, GF6, Hahnemühle Fineart, Germany) positioned 57 cm above the burner, with vacuum assistance (Seco SV 1025 C, Busch, Switzerland). The collected powder was retrieved by scraping the filter with a spatula, followed by sieving through a 250 µm stainless steel mesh to eliminate residual filter fibers. Obtained powders were annealed at 500°C and air atmosphere in an oven (CWF 1300, Carbolite Gero, Germany) before characterizations and gas sensor testing.

### 4.2 Synthesis of Cu_1_‐Co_3_O_4_

Wet impregnation [21, 26] was used to stabilize Cu single‐atoms onto the pure, unannealed Co_3_O_4_ nanoparticles. 100 mg of pure FSP‐made and non‐annealed Co_3_O_4_ nanoparticles and 36.6 mg of Cu(NO_3_)_2_∙2.5H_2_O (ACS reagent, 98%, Sigma–Aldrich, Switzerland) were dispersed in 10 mL deionized water. The dispersion of the nanoparticles in aqueous Cu(NO_3_)_2_ solution was vigorously stirred with a magnetic stirrer for 6 h before being washed and centrifuged several times with DI water and ethanol. Powders collected after centrifugation were dried in an oven overnight at 70°C, then annealed in a furnace at 500°C for 5 h under static air.

### 4.3 Synthesis of CuO_NP_‐Co_3_O_4_

First, pure CuO nanoparticles were prepared using the same protocol with the FSP reactor, except that the precursor solution is composed of 0.2 m of Deca Copper 8 (Borchers, Germany) in xylene. Also, the feeding rate of the precursor solution was reduced to 2 mL min^−1^. To prepare CuO_NP_‐Co_3_O_4_ nanoparticles, the same protocol was used as for the wet impregnation in the synthesis of Cu_1_‐Co_3_O_4_, by replacing the Cu(NO_3_)_2_ precursor with CuO nanoparticles with the same atomic loading of Cu (1.42 wt.%).

### 4.4 Chemoresistive Gas Sensor Preparation and Measurement

6 mg of annealed pure Co_3_O_4_, Cu_1_‐Co_3_O_4,_ and CuO_NP_‐Co_3_O_4_ were each dispersed in 120 µL of ethanol by ultrasonication. Dispersed solutions were each completely dropped onto alumina substrates with interdigitated electrodes and a back heater (electrode type #103, Electronic Design Center, Case Western University, USA), and spin‐coated at 250 RPM for 30 min. The resulting films were dried in an oven at 70°C for 30 min.

The sensors were affixed to Macor holders and positioned within a Teflon chamber, as detailed in previous studies [91]. The operating temperature was regulated by applying a constant voltage to the Pt heater embedded in the alumina sensing substrate. The chamber was integrated into a gas mixing system [92] via inert Teflon tubing. Hydrocarbon‐free synthetic air (PanGas, C_x_H_y_ and NO_x_ < 100 ppb, Switzerland) was employed as the carrier gas. Certified gas mixtures (PanGas, Switzerland) were blended using calibrated mass flow controllers (Bronkhorst, Netherlands) to achieve the target composition. The gas standards included: acetone (14.9 ppm, PanGas, Switzerland), toluene (9.6 ppm, PanGas, Switzerland), ethanol (15.0 ppm, PanGas, Switzerland), ammonia (10.3 ppm, PanGas, Switzerland), methane (10.2 ppm, all in synthetic air, Pangas, Switzerland), NO (10.2 ppm, PanGas, Switzerland), acetaldehyde (17.1 ppm, PanGas, Switzerland), and formaldehyde (17.0 ppm all in N_2_, PanGas, Switzerland). To generate humidified air, dry synthetic air was passed through a bubbler containing deionized water at room temperature (ca. 25°C), and the resulting moisture‐laden stream was blended with the analyte‐containing gas flow to achieve the target relative humidity. The total gas flow rate was maintained at 300 mL min^−1^. The dynamic ohmic resistance of the sensing film deposited on the electrodes was monitored using a digital multimeter (Keithley 2700, Keithley Instruments, USA). The chemoresistive sensor response S was defined by the equation below:

$$S=\frac{R_{g}}{R_{a}}\;-1$$

where R_g_ and R_a_ are the resistances of the sensing layer under gas exposure and in air, respectively. The signal‐to‐noise ratio (SNR) of the Cu_1_‐Co_3_O_4_ sensor at 5 ppb formaldehyde was evaluated as:

$$SNR=\frac{\Delta R}{Noise}$$

where ΔR is the change in resistance upon exposure to 5 ppb of formaldehyde and the noise is the standard deviation of the baseline readout over 3 min prior to exposure.

The theoretical lower detection limit of the Cu_1_‐Co_3_O_4_ sensor is calculated by extrapolating SNR versus formaldehyde gas concentration, assuming that the SNR changes linearly with gas concentration:

$$\frac{5\;ppb}{SNR_{5\;ppb}}=\frac{c_{LOD}}{SNR_{LOD}}$$

where c_LOD_ is the lower detection limit and SNR_LOD_ is the lowest acceptable SNR at the lower detection limit, which is typically set as 3.

### 4.5 Materials Characterization

XRD patterns of powders were acquired with a Bruker D2 Phaser (USA) operated at 30 kV and 10 mA (Cu K_α_ radiation, λ = 1.5406 Å), with a scanning step size of 0.01° and a scanning time of 2.2 s per step. All XRD patterns were corrected for sample displacement using crystalline tin telluride (SnTe, 99.999%, Sigma Aldrich, Switzerland) as an internal standard [93]. To this end, all samples were ground with SnTe in a mortar, and the resulting XRD pattern was aligned to the reference reflections of cubic SnTe (PDF 46–1210).

X‐ray absorption spectroscopy measurements were taken at the Balder beamline of MAX IV (Lund, Sweden) with transmission and fluorescence XANES and EXAFS in the energy range from 8–10 keV (double crystal monochromator Si(111)) at room temperature. Samples were prepared as pressed pellets (13 mm diameter) by homogenizing the catalyst with boron nitride (1:5 mass ratio) to minimize self‐absorption effects. Analyses of the near edge and extended range were carried out using Athena software, and the fitting of each sample was carried out by Artemis software. EXAFS spectra were fitted in a Fourier‐transform range of 3–11 Å^−1^ with a Hanning window applied between 1 and 3 Å. NEXAFS measurements were carried out under vacuum at the B07 beamline of Diamond Light Source (UK). Reference samples (CuO, 99.99% trace metal basis, and Cu_2_O, ≥99.99% trace metals basis, anhydrous) were purchased from Sigma–Aldrich, Switzerland. XPS was carried out using Al Kα radiation (Sigma Probe, Thermo VG Scientific, USA). Surface morphology and cross‐section SEM images of the nanoparticles were carried out by field‐emission SEM under 2.0 kV and 0.10 nA (Thermofisher Scientific, Magellan 400, USA).

Particle characterization was carried out using transmission electron microscopy (TEM) and scanning transmission electron microscopy (STEM) on a JEOL JEM‐F200 equipped with a cold field emitter as electron source, operated at 200 kV (JEOL, Japan). Samples for imaging were prepared by dispersing the particles in ethanol and depositing them onto perforated carbon films supported on molybdenum grids. For compositional analysis, energy‐dispersive X‐ray spectroscopy (EDXS) was performed in STEM mode utilizing four silicon drift detectors (SDDs) from JEOL. High‐resolution TEM (HR‐TEM) imaging was conducted on a Grand ARM300F microscope (JEOL, Japan) operating at 300 kV with a cold field emission source, where aberration correction for both the image‐forming and probe‐forming lenses allowed sub‐angstrom resolution in TEM and STEM modes. Quantitative analysis of Cu loading was performed by inductively coupled plasma mass spectrometry (ICP–MS, Agilent 7900, Agilent Technologies, USA). Samples (∼5 mg) were digested in a mixture of HNO_3_ and HCl using microwave‐assisted acid digestion (overnight) prior to measurement.

Temperature‐programmed experiments were performed with an Autochem III chemisorption analyzer (Micromeritics, USA), equipped with a TCD detector and connected to a quadrupole mass spectrometer (Omnistar, Pfeiffer, Germany). For H_2_‐TPR, approx. 40 mg of powders were pretreated under Ar at 200°C. The reduction was performed under 30 mL/min of 5 vol% H_2_ in Ar, between 40°C and 600°C at a rate of 5°C/min. A moisture trap ensured that no humidity reached the TCD, as also confirmed by mass spectrometry. For formaldehyde‐TPD, approx. 60 mg of powders were pretreated in He at 200°C. Formaldehyde (10 ppm in N_2_, Pangas, Switzerland) was supplied at a rate of 10 mL/min at 30°C for 120 min. Thereafter, desorption was carried out under 10 mL/min He between 30°C and 600°C at a rate of 5°C/min, and analyzed by mass spectrometry at m/z ratios of 2, 18, 28, 29, 30, 44.

Catalytic oxidations of formaldehyde over Cu_1_‐Co_3_O_4_ and CuO_NP_‐Co_3_O_4_ were performed using the method reported in previous literatures [40, 94]. 10.2 mg of catalyst powder was packed in a glass tube between quartz wool plugs and heated in a horizontal furnace (Carbolite ESZ 12/450, Germany). The reactor was supplied with the same gas‐mixing system used for sensing at a total flow of 150 mL min^−1^ under dry air, and a formaldehyde concentration of 1 ppm. The bed was heated from 20°C to 250°C with a ramp rate of 10°C min^−1^, including 1 h isothermal holds at each temperature for reaction stabilization. Outlet concentrations were analyzed online with a PTR‐TOF‐MS (Ionicon PTR‐ToF‐MS 1000, Austria) operated with H_3_O^+^ primary ions at 600 V, 60°C, 2.3 mbar, and a reduced field of 130 Td. The applied conditions corresponded to a weight hourly space velocity of ∼0.88 mL_formaldehyde_ h^−1^ g_cat_^−1^. Characteristic m/z signals were monitored at 31.02 (formaldehyde) [95]. Instrument calibration was performed daily with five‐point standards between 0 and 1000 ppb with a gas standard.

The catalytic conversion is defined as:

$$\chi =1-\frac{c_{out}\;\left(ppm\right)}{c_{in}\;\left(ppm\right)}$$

where χ is the catalytic conversion, c_out_ and c_in_ are the concentration of formaldehyde at the exhaust and concentration of formaldehyde injected, respectively. Assuming pseudo‐first‐order kinetics at ppm‐level analyte concentrations [96], the reaction rate r normalized to catalyst mass was determined by:

$$r\;\left(\frac{mol}{g\;s}\right)=\frac{Q_{total}\;\left(m^{3}\;s^{-1}\right)\;c_{in}\;\left(\frac{mol}{m^{3}}\right)\ln\left(\frac{1}{1-\chi }\right)}{m_{cat}\;\left(g\right)}$$

where Q_total_ is the total inlet flow and m_cat_ is the catalyst mass. The apparent activation energy E_a_ is obtained from Arrhenius analysis of the temperature‐dependent r according to:

$$r\;=\;A\;e^{-\frac{E_{a}}{RT}}$$

where A is the pre‐exponential constant, R (kJ mol^−1^ K^−1^) is the universal gas constant and T (K) is the temperature.

### 4.6 DFT Simulation

All spin‐polarized DFT+U calculations were performed using the Vienna Ab initio Simulation Package (VASP) [97, 98] to examine the interaction of formaldehyde with pristine Co_3_O_4_, CuO_NP_‐Co_3_O_4_, and Cu_1_‐Co_3_O_4_. The projector augmented‐wave (PAW) method and the Perdew–Burke–Ernzerhof (PBE) functional within the generalized gradient approximation (GGA) were used [99]. The on‐site Hubbard correction for Co was set to U = 4.4 eV, following the previous DFT study on formaldehyde adsorption on Co_3_O_4_ to ensure methodological consistency and enable direct comparison [100]. A plane‐wave cutoff of 400 eV was used. Structural optimizations were performed until the total‐energy change was below 1 × 10^−4^ eV and the residual forces were smaller than 0.04 eV/Å. Brillouin‐zone sampling for slab models was carried out using a 3 × 2 × 1 Monkhorst–Pack k‐point mesh.

### 4.7 Surface and Interface Models

The bulk Co_3_O_4_ structure was constructed based on experimental data (JCPDS No. 42–1467). Surface calculations were carried out using four‐layer Co_3_O_4_ (110) slabs with a commonly adopted termination in prior computational studies [100, 101]. A vacuum spacing of 15 Å was introduced to avoid interactions between periodic images. The CuO_NP_‐Co_3_O_4_ model was constructed by extending the Co_3_O_4_ slab along a direction and adding a two‐layer CuO nanowire, following the methodology described for ZnO/Cu systems [102]. Additionally, the Cu_1_‐Co_3_O_4_ model was generated by substituting one surface Co atom with a Cu atom, a standard strategy to represent surface single‐atom sites on spinel oxides, as previously reported for other substituted single‐atoms (e.g., W and Ru) [103, 104] and consistent with recent modeling of Cu SAs on Co_3_O_4_ surfaces [105]. To evaluate formaldehyde adsorption, various molecular orientations and sites were analyzed across all systems. For all three systems, the bottom half of the slab layers was fixed to mimic bulk constraints, while all other atoms were fully relaxed.

### 4.8 Oxygen‐vacancy formation energy

The oxygen‐vacancy formation energy (*E_vf_*) on pure Co_3_O_4_ and Cu_1–_Co_3_O_4_ was defined as follows:

$$E_{vf}=E_{defect}\;-E_{perfect}+\mu_{O}$$

where *E_defect_*, *E_perfect_* are the total energies of the defective and stoichiometric (110) surfaces, respectively. The oxygen chemical potential was taken as $\mu_{O}=\frac{1}{2}\;E\left(O_{2}\right)$, with *E*(*O*_2_) being the total energy of an isolated O_2_ molecule.

### 4.9 Adsorption Energy

The adsorption energy of formaldehyde (*E_ad_*) was calculated as follows:

$$E_{ad}=E_{slab+HCHO}\;-E_{slab}-E_{HCHO}$$

where *E*_*slab* + *HCHO*_ is the energy of the adsorbed system, *E_slab_* and *E_HCHO_* are the energies of the clean surfaces and the isolated HCHO molecule, respectively.

### 4.10 Charge Analysis

Bader charge analysis [106, 107] and charge density difference (CDD) were also performed to study the influence of SA‐Cu on the Co_3_O_4_. The CDD was defined as:

$$\Delta \rho =\rho_{Cu1-Co3O4}\;-\rho_{Cu1}-\rho_{Co3O4}$$

where ρ_*Cu*1 − *Co*3*O*4_, ρ_*Cu*1_ and ρ_*Co*3*O*4_ represent the charge densities of the Cu_1_‐Co_3_O_4_ system, the isolated Cu atom, and the Co_3_O_4_ slab, respectively. Crucially, to ensure a meaningful subtraction, the charge densities of the isolated components ρ_*Cu*1_ and ρ_*Co*3*O*4_ were obtained through single‐point calculations using the same supercell dimensions and atomic coordinates as the optimized ρ_*Cu*1 − *Co*3*O*4_ structure, without further geometry relaxation.

### 4.11 Statistical Methods

The average crystallite sizes of the Co_3_O_4_‐based samples were estimated using the Scherrer equation, applied to the 311, 511, and 440 diffraction planes identified in the XRD patterns. The full width at half maximum (FWHM) values were extracted after background subtraction and instrumental broadening correction. The crystallite sizes calculated from the three reflections are indicated as average and standard deviation to obtain a representative value for each sample.

XPS spectra were processed and fitted using the Thermo Scientific Avantage software. Background subtraction was performed using the Shirley method, which is the standard approach in Avantage for core‐level spectra and accounts for inelastic background contributions. In cases where the Shirley background was not applicable due to spectral shape, a linear background was used as implemented in Avantage. Peak fitting was carried out using a non‐linear least squares algorithm with a combination of Gaussian–Lorentzian (Voigt) peak shapes, allowing peak positions, widths, and intensities to vary within physically reasonable limits. All processing steps, including background subtraction and peak fitting, were recorded in the Avantage audit trail for reproducibility. Quantification was based on the integrated peak areas after background subtraction, applying the instrument transmission function and appropriate sensitivity factors as provided by the Avantage library. For the chemical speciation, we based it on multiplet‐constrained deconvolution and complementary reducibility probes rather than on apparent shifts of the peak maxima, which are not reliable indicators of small chemical changes under these conditions [108, 109].

EXAFS fitting was performed on the Cu K‐edge spectra using the Artemis software package. The theoretical paths were generated from the FEFF6 code based on a Co_3_O_4_ lattice model with Cu substitution. For Cu_1_‐Co_3_O_4_, fitting was carried out using a two‐shell model: a Cu–O shell with a coordination number of 3.5 and a Cu–Co shell at ∼2.89 Å with CN ≈ 1. No Cu–Cu scattering was included, consistent with the absence of Cu clustering. Reference compounds (CuO, Cu_2_O, Cu foil) were also fitted for comparison. Good agreement between experimental and fitted spectra is shown in Figure S9.

## Conflicts of Interest

The authors declare no conflicts of interest.

## Supporting information




**Supporting File**: smll73370‐sup‐0001‐SuppMat.docx.

## Data Availability

The data that support the findings of this study are available from the corresponding author upon reasonable request.
