# Supplementary material for: Single Cu Atom Sites on Co3O4 Activate Interfacial Oxygen for Enhanced Reactivity and Selective Gas Sensing at Low Temperature
Source: Small. 2026 Apr 13;22(32):e00033. doi: 10.1002/smll.202600033 (PMC13244293; doi:10.1002/smll.202600033)
Supplement: Supplementary file 1 — Supporting File: smll73370‐sup‐0001‐SuppMat.docx. [file SMLL-22-e00033-s001.docx]

# Supplementary Information

# Single Cu Atom Sites on Co_3_O_4_ Activate Interfacial Oxygen for Enhanced Reactivity and Selective Gas Sensing at Low Temperature

Hamin Shin^1^, Matteo D’Andria^1^, Meng Yin^2^, Jaehyun Ko^1^, Ken Suzuki^2^, Dong-Ha Kim^3^, Frank Krumeich^4^, Andreas T. Güntner^1^*

^1^Human-centered Sensing Laboratory, Department of Mechanical and Process Engineering, ETH Zürich, CH-8092, Zürich, Switzerland

^2^Green X-Tech Center, Green Goals Initiative, Tohoku University, Sendai, Miyagi 980-8579, Japan

^3^Department of Materials Science and Chemical Engineering, Hanyang University, Ansan 15588, Republic of Korea

^4^Department of Chemistry and Applied Biosciences, Laboratory of Inorganic Chemistry, ETH Zürich, CH-9083 Zürich, Switzerland

^*^corresponding author: [andregue@ethz.ch](mailto:andregue@ethz.ch)


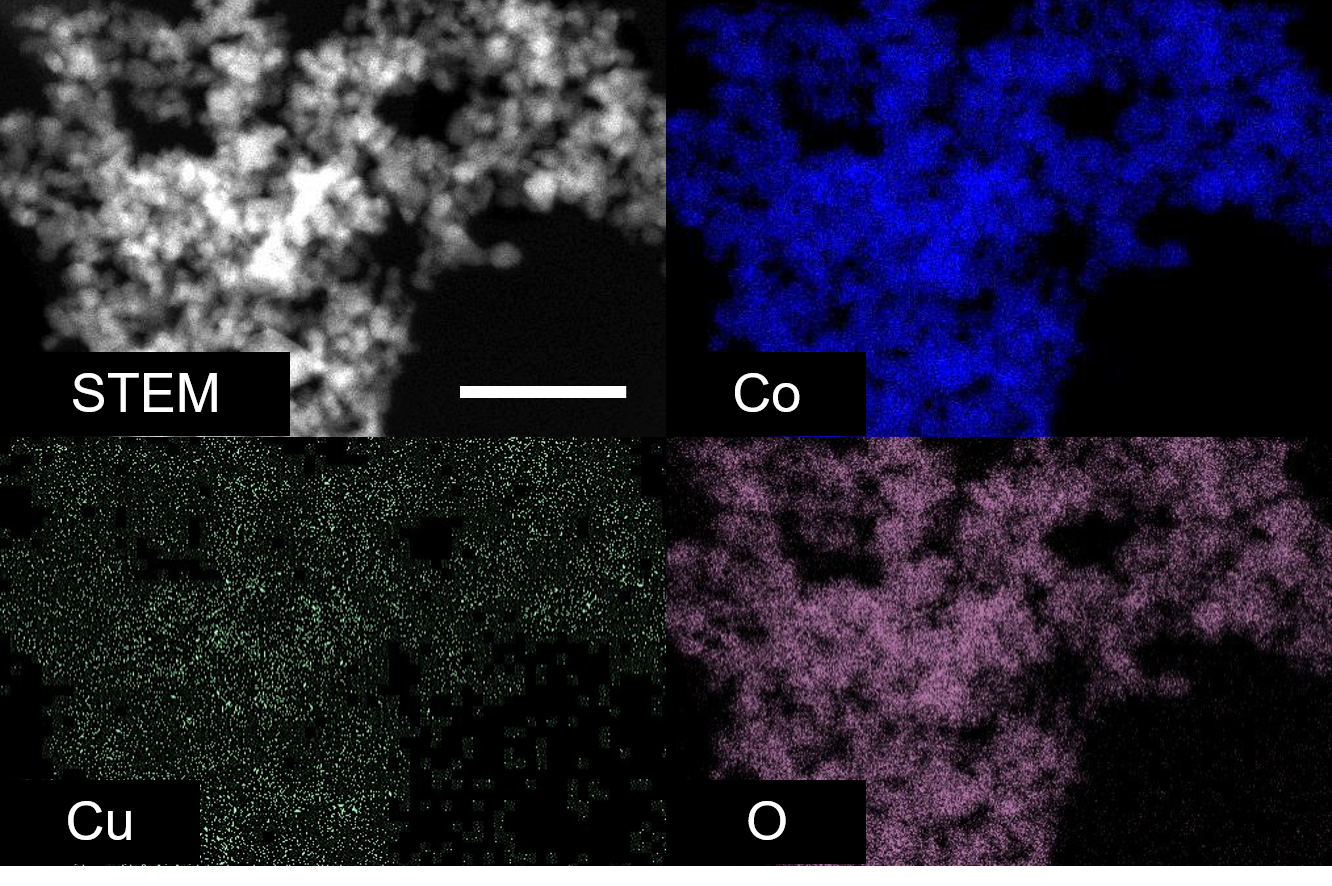


Figure S1: STEM image of Cu_1_-Co_3_O_4_ and corresponding EDS mapping of Co, Cu, and O. Scale bar: 250 nm.


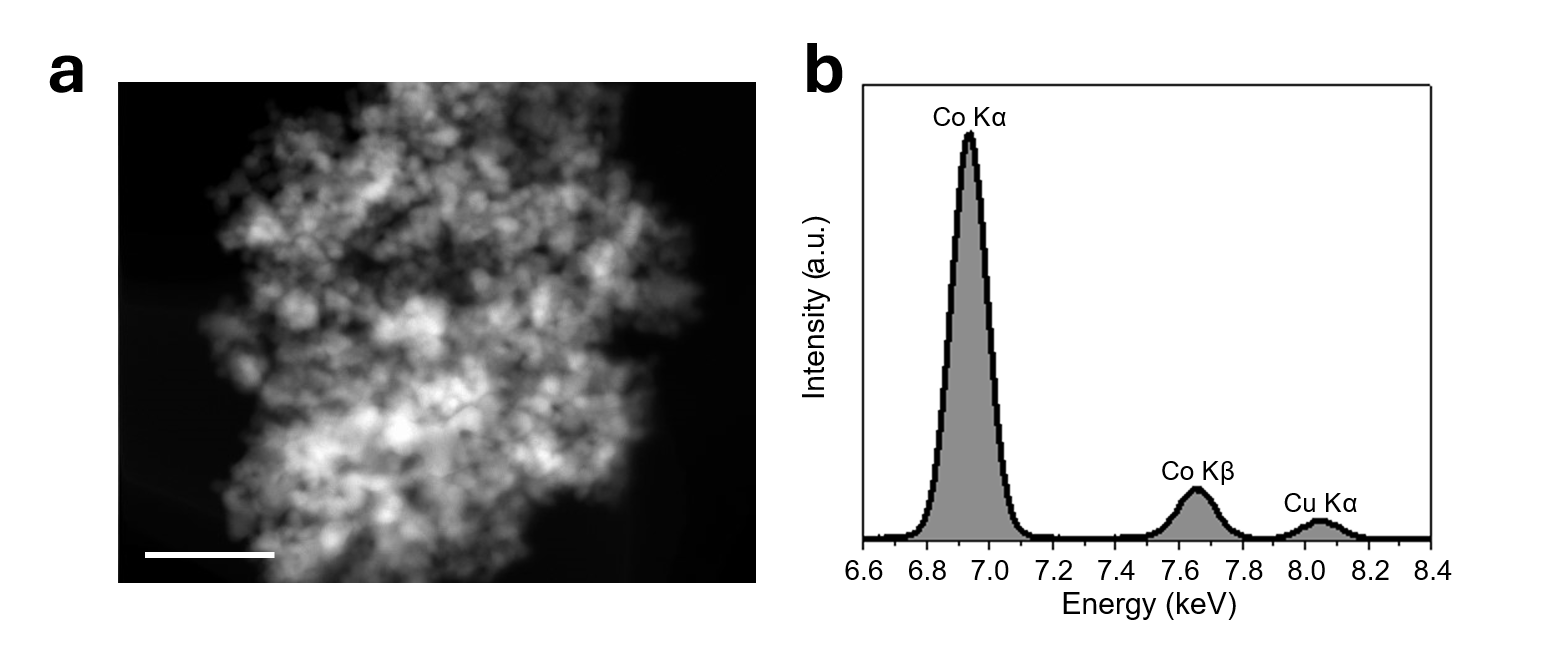


Figure S2: a) Scanning transmission electron microscopy (STEM) image of Cu_1_-Co_3_O_4_ nanoparticles. Scale bar: 200 nm. b) Energy-dispersive X-ray spectroscopy (EDS) spectrum collected from (a), confirming the elemental composition.

**
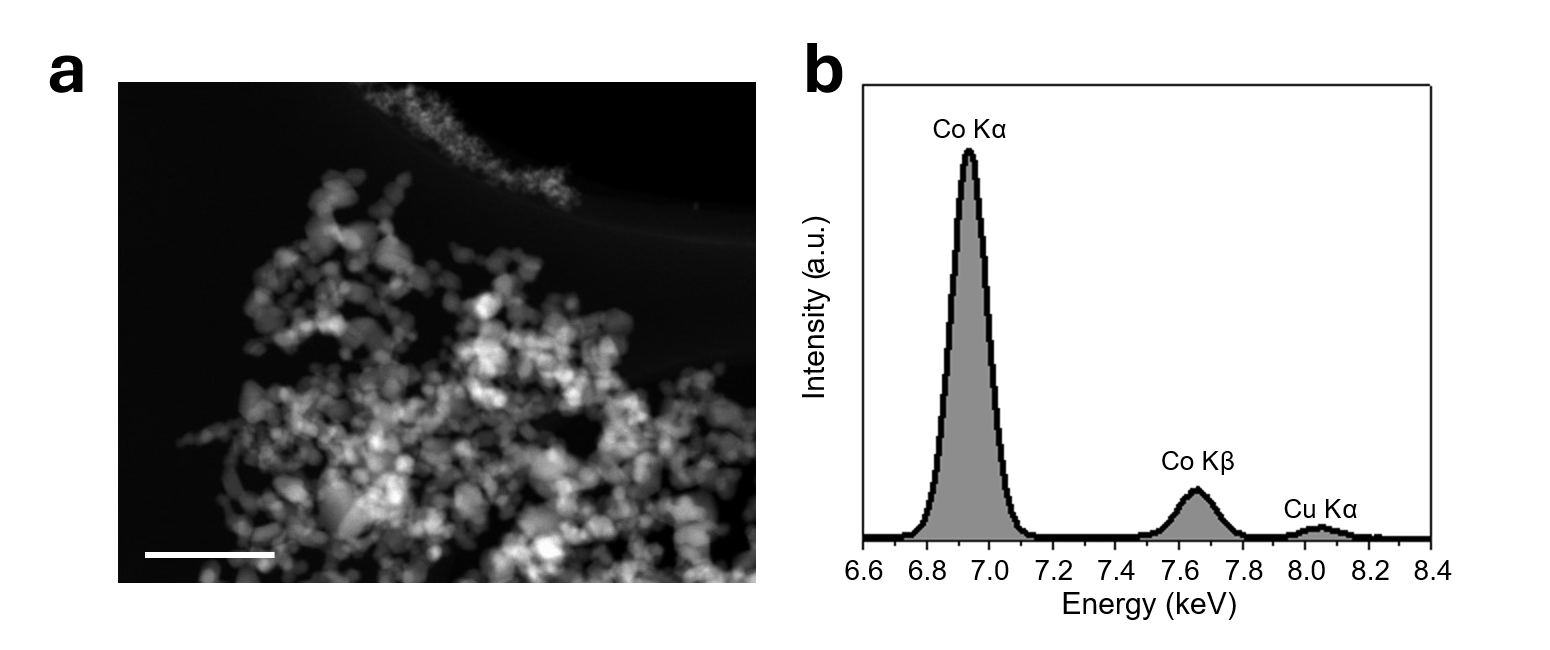
**

Figure S3: a) Scanning transmission electron microscopy (STEM) image of Cu_NP_-Co_3_O_4_ nanoparticles. Scale bar: 200 nm. b) Energy-dispersive X-ray spectroscopy (EDS) spectrum collected from (a), confirming the elemental composition.


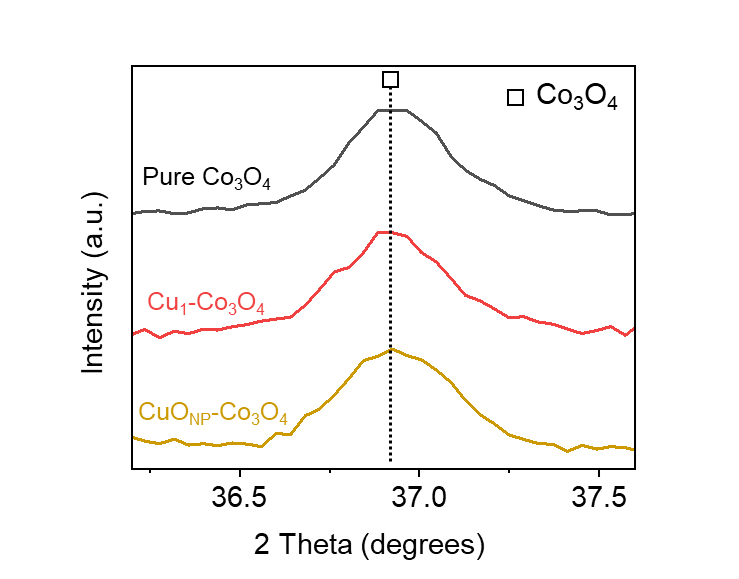


**Figure S4:** XRD patterns of pure Co_3_O_4_, Cu_1_-Co_3_O_4_, and CuO_NP_-Co_3_O_4_ between 2 theta = 36.2 – 37.8°. Indicated square is the reference peak of cubic Co_3_O_4_. The patterns are corrected for sample displacement with an internal standard, SnTe.^1^


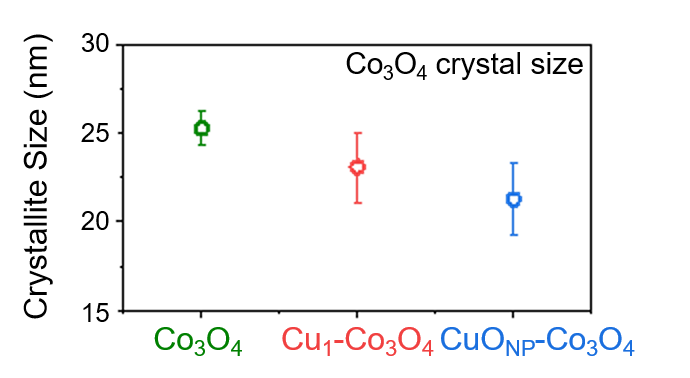


Figure S5: Co_3_O_4_ Crystallite sizes of pure Co_3_O_4_, Cu_1_-Co_3_O_4_, and CuO_NP_-Co_3_O_4_. Symbols indicate average and error bars standard deviation of the crystal size estimated by the Scherrer equation from the (311), (511), and (440) planes.


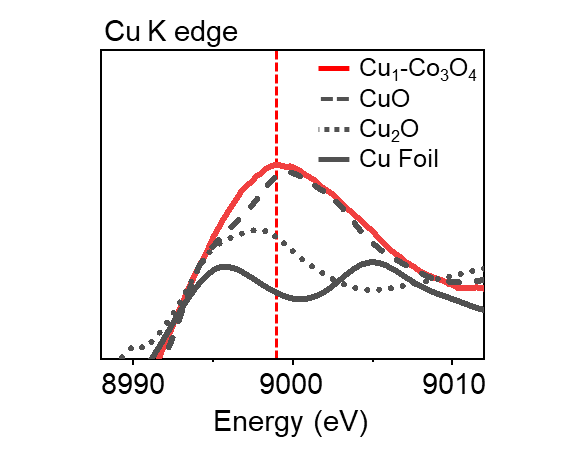


Figure S6: Magnified XANES spectra of Cu_1_-Co_3_O_4_ and reference samples at Cu K edge. The red dashed vertical line indicates the position of the absorption peak of Cu species in Cu_1_-Co_3_O_4_.


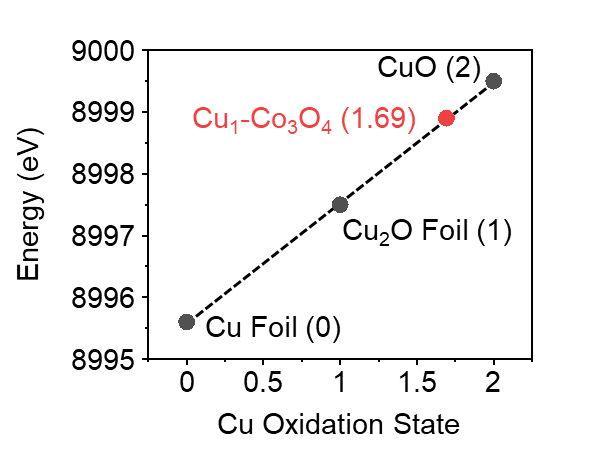


Figure S7: Linear interpolation of Cu K-edge XANES edge positions using Cu foil (Cu oxidation state = 0), Cu_2_O (+1), and CuO (+2) as references, giving an average Cu oxidation state of +1.69 for Cu_1_-Co_3_O_4_.


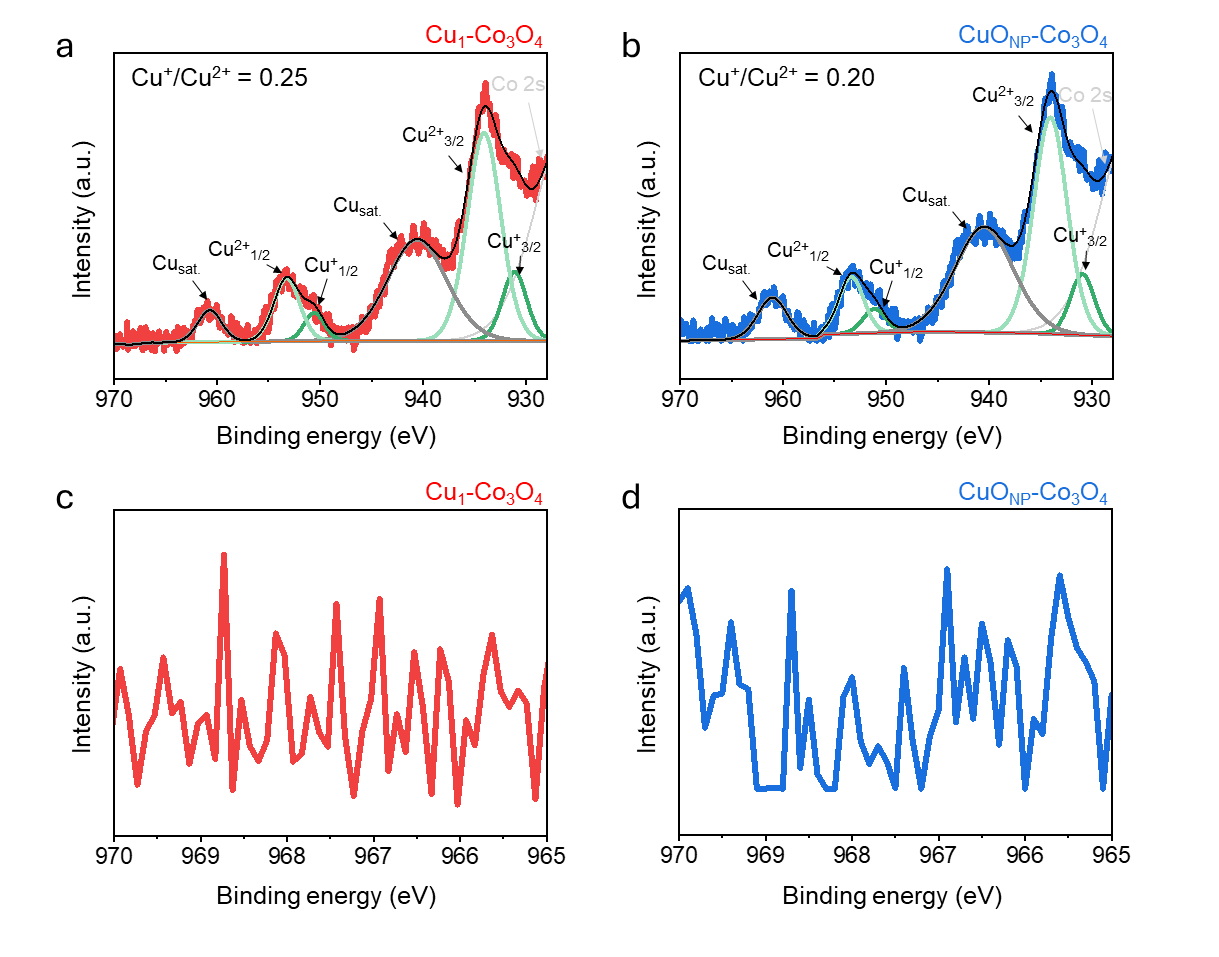


**Figure S8.** Cu 2p XPS spectra and noise feature (970-965 eV) of a, c) Cu_1_-Co_3_O_4_, and b, d) CuO_NP_-Co_3_O_4_. Co 2s peak is present due to dominance of Co_3_O_4_. Relative peak area ratio between Cu^+^ and Cu^2+^ is determined to be 0.25 and 0.20 for Cu_1_-Co_3_O_4_ and CuO_NP_-Co_3_O_4_, respectively.


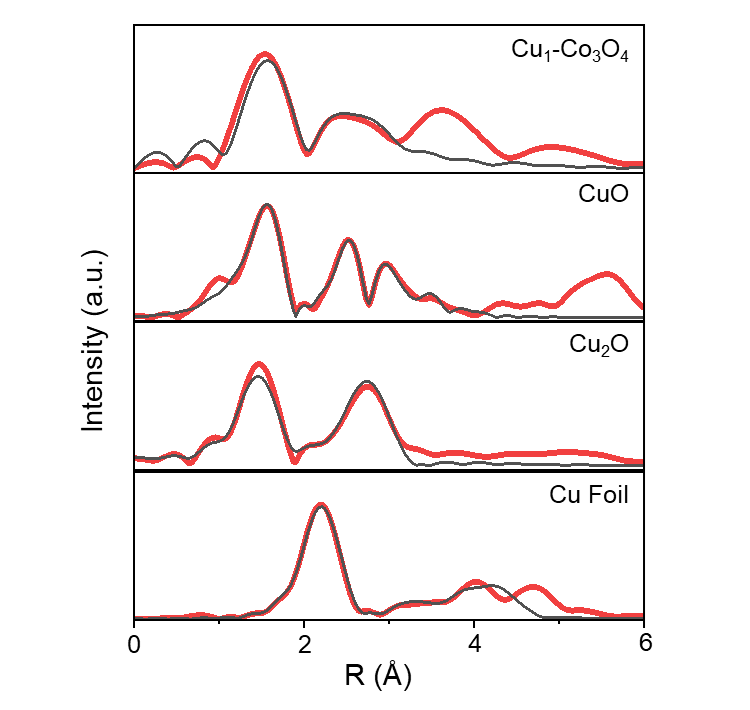


Figure S9: EXAFS curve fittings of Cu_1_-Co_3_O_4_ and reference samples. Red lines are the experimental data, and black lines are the fit.


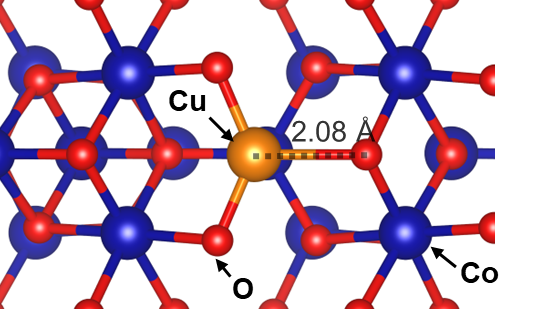


Figure S10: Alternative view of the atomic model of Cu SA anchored on Co_3_O_4_ surface.


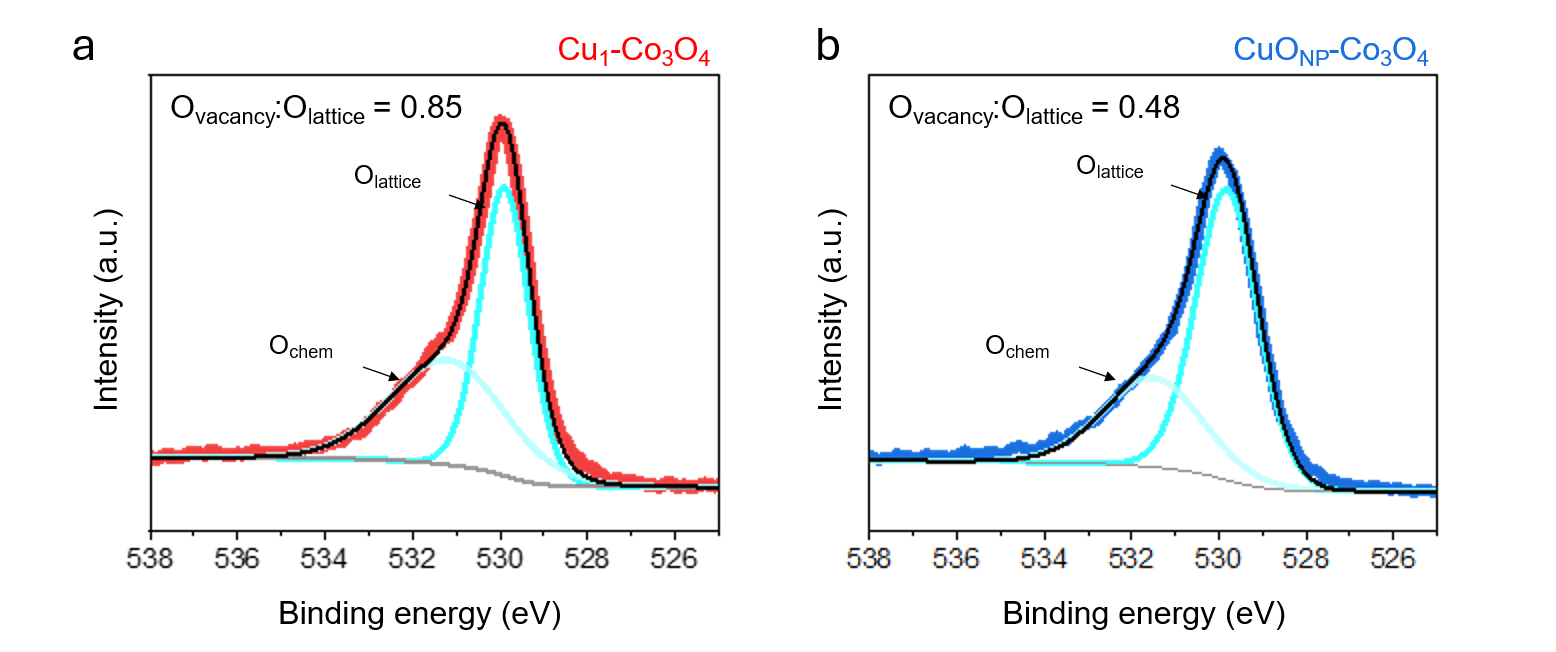


**Figure S11:** O 1s XPS spectra of Cu_1_-Co_3_O_4_ and CuO_NP_-Co_3_O­. Cu_1_-Co_3_O_4_ exhibits a significantly higher vacancy-related (O_chem_) to lattice oxygen (O_lattice_) peak area ratio (0.85) compared to CuO_NP_-Co_3_O_4_ (0.48).


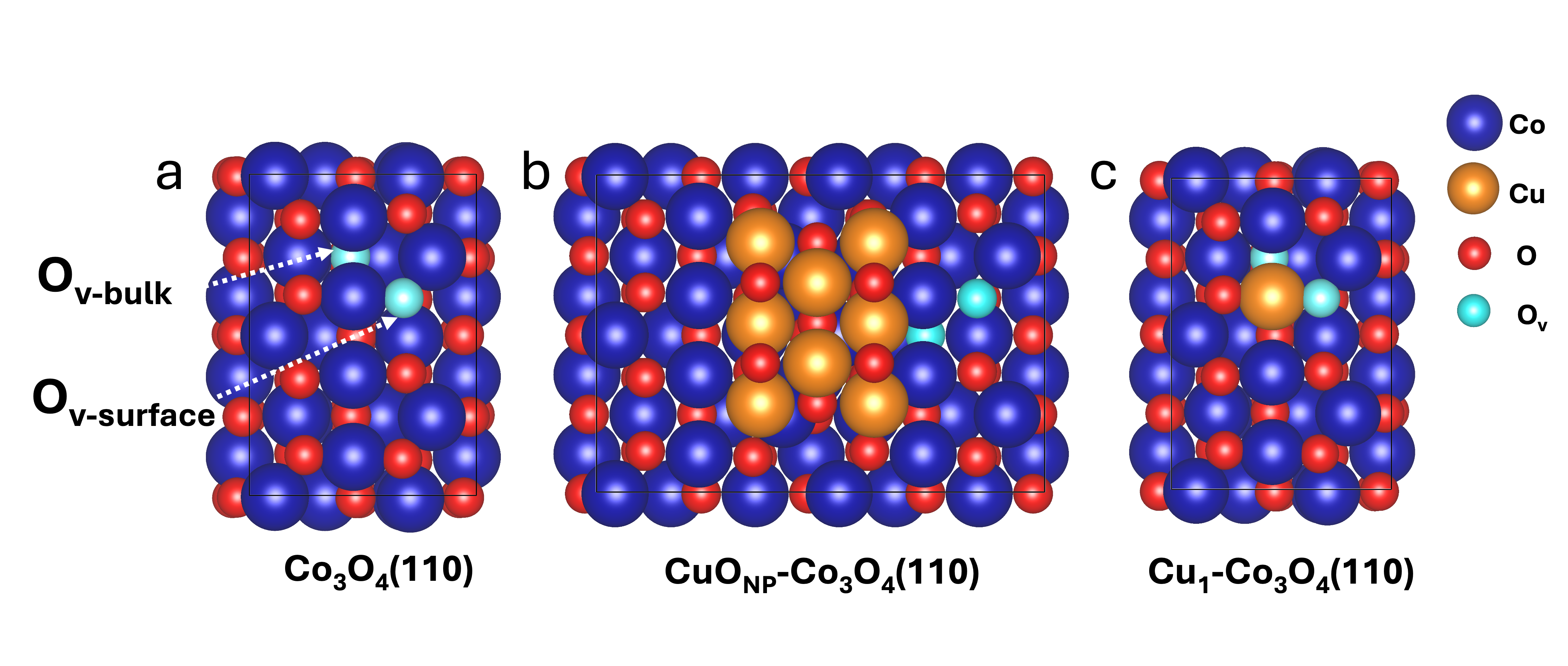


**Figure S12:** Atomic model as well as the position of the oxygen vacancies in bulk and surface sites for a) Co_3_O_4_, b) CuO_NP_-Co_3_O_4_ and c) Cu_1_-Co_3_O_4_ slab model.


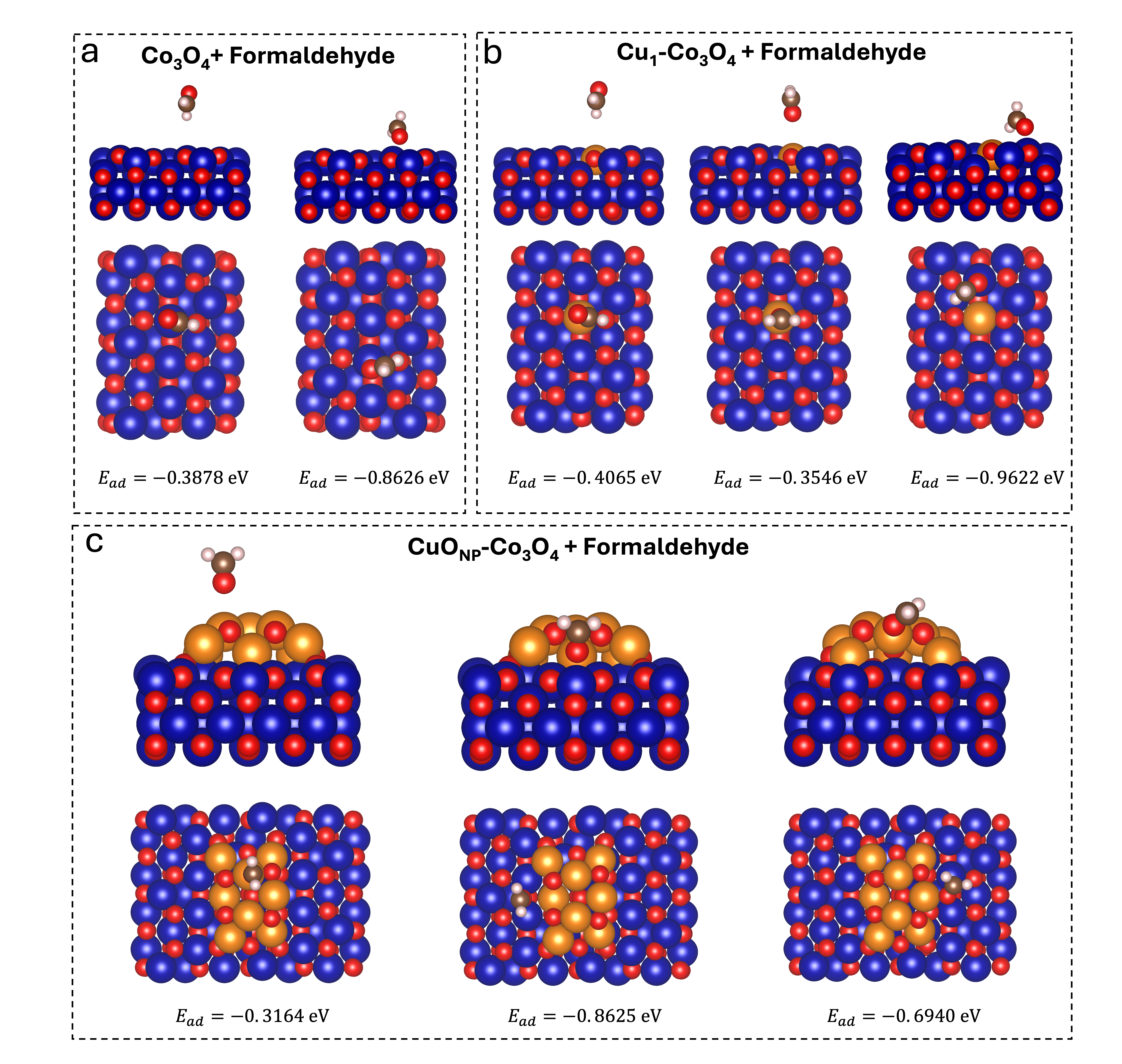


**Figure S13:** Various formaldehyde adsorption configurations and the corresponding calculated adsorption energies, $E_{ad}$. a) Two adsorption configurations for the Co_3_O_4_ surface: the H atom of formaldehyde points to a Co atom on the surface; the O atom of formaldehyde points to a Co atom on the surface. b) Three configurations for the Cu_1_-Co_3_O_4_(110) surface: H atom pointing to the SA-Cu site, O atom pointing to the SA-Cu site, and O atom pointing to a neighboring Co site. c) Three configurations for the CuO_NP_-Co_3_O_4_: O atom of formaldehyde adsorption on a Cu site from the CuO_NP_, on the edge site at the CuO_NP_-Co_3_O_4_ boundary, and on a Co atom from the Co_3_O_4_ surface.


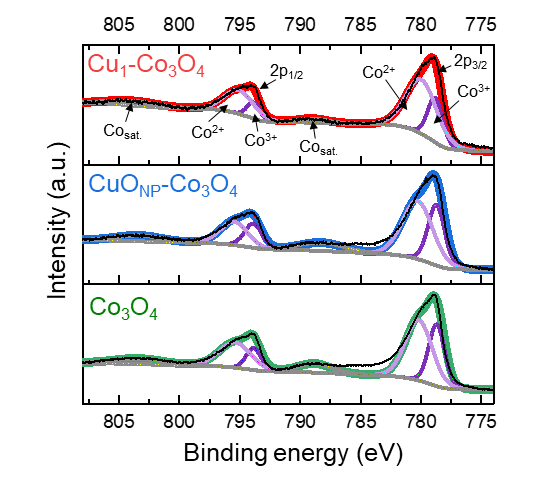


Figure S14: XPS spectra of Cu_1_-Co_3_O_4_, CuO_NP_-Co_3_O_4_, and Co_3_O_4_ in the vicinity of Co 2p. Black line is the experimental data, and the red, blue and green lines are the fit data for Cu_1_-Co_3_O_4_, CuO_NP_-Co_3_O_4_ and pure Co_3_O_4_, respectively. Light purple and dark purple are the Co^2+^ and Co^3+^ peak fits, respectively.


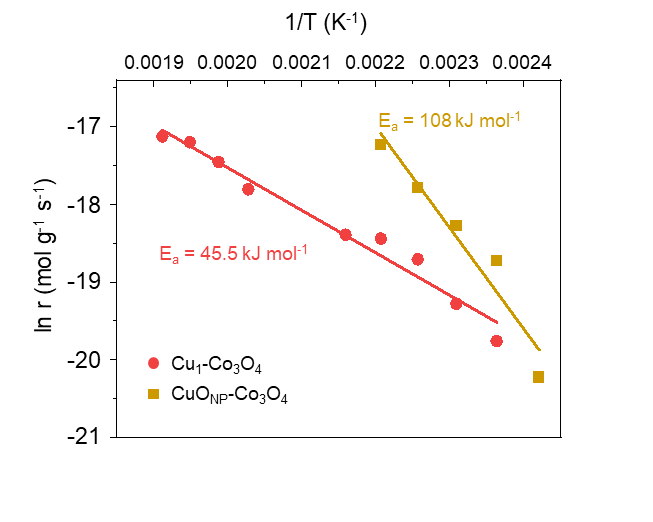


**Figure S15:** Arrhenius plots of the pseudo-first-order rate normalized per catalyst mass, yielding apparent activation energies of 45.5 kJ mol^-1^ (Cu_1_-Co_3_O_4_) and 108 kJ mol^-1^ (CuO_NP_-Co_3_O_4_). See Experimental for details.

**
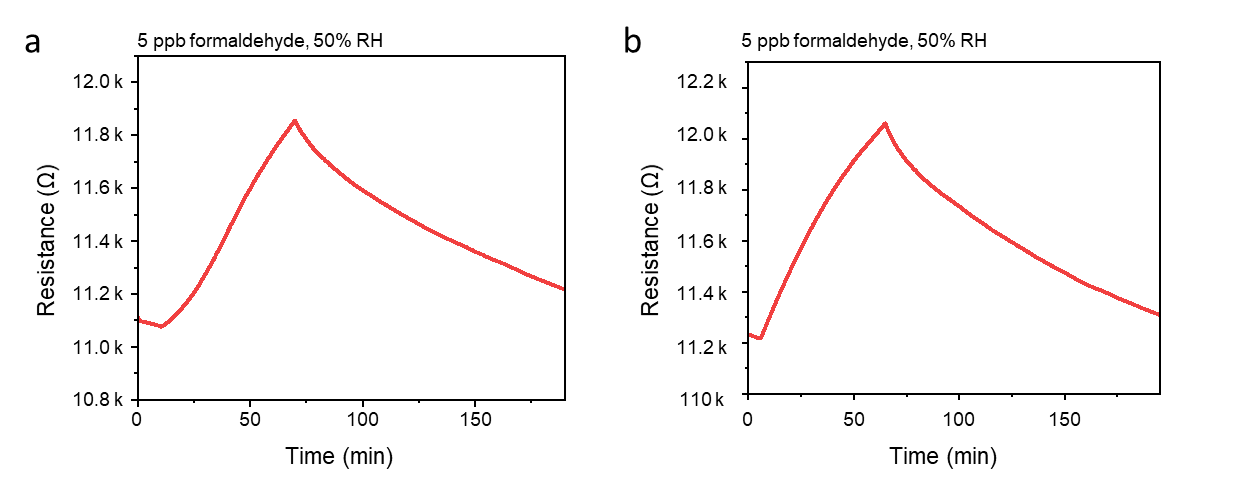
**

**Figure S16:** Validation of reproducibility at the detection limit of 5 ppb. Dynamic response of two independently fabricated Cu_1_-Co_3_O_4_ sensors toward 5 ppb formaldehyde at 75 °C. Both sensors exhibit consistent response (~0.07), confirming reproducible detection performance in agreement with Figure 4c.


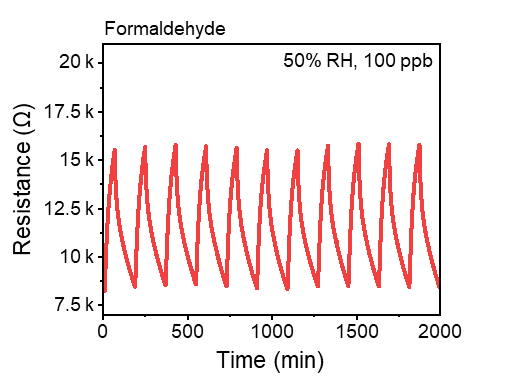


Figure S17: Stability of Cu_1_-Co_3_O_4_ under 11 repeated exposure and recovery to 100 ppb formaldehyde.


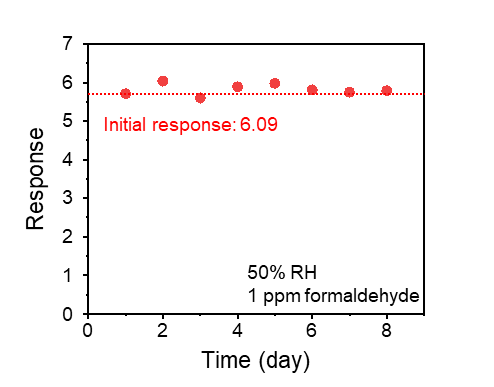


**Figure S18:** Long-term stability of Cu_1_-Co_3_O_4_ sensor toward 1 ppm formaldehyde at 75 °C over a period of 8 days.


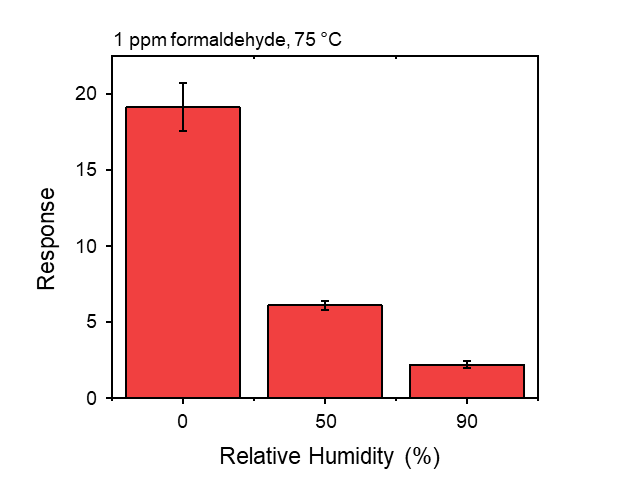


**Figure S19:** Effect of humidity on Cu_1_-Co_3_O_4_ sensor response toward 1 ppm formaldehyde at 75 °C under 0%, 50%, and 90% RH.

Table S1: EXAFS fitting table. CN: coordination number, R: bond distance, σ^2^: Debye-Weller factor.

| **Sample** | **Shell** | **CN** | **R (Å)** | **σ^2^ (Å^2^)** | **R-factor** |
| --- | --- | --- | --- | --- | --- |
| Cu_1_-Co_3_O_4_ | Cu–O | 3.5 | 2.08±0.051 | 0.037±0.029 | 0.009 |
|  | Cu–Co | 1 | 2.89±0.023 | 0.007±0.016 |  |
| CuO | Cu–O | 4 | 1.95±0.004 | 0.005±0.001 | 0.006 |
|  | Cu–Cu | 4 | 2.94±0.008 | 0.005±0.001 |  |
| Cu_2_O | Cu–O | 2 | 1.85±0.013 | 0.005±0.003 | 0.019 |
|  | Cu–Cu | 12 | 3.02±0.016 | 0.020±0.002 |  |
| Cu Foil | Cu–Cu | 12 | 2.54±0.001 | 0.009±0.001 | 0.006 |

**Table S2.** Local Bader charge and electron transfer values for the single-atom Cu in the Cu_1_-Co_3_O_4_ model (Negative values ΔQ mean that the atom loses electrons).

| **Cu_1_-Co_3_O_4_** | **Bader Charge (q)/e^-^** | $\boldsymbol{\Delta}$**Q/e^-^** |
| --- | --- | --- |
| Cu | 10.378 | -0.622 |
| O_1_ | 6.882 | 0.882 |
| O_2_ | 7.075 | 1.075 |
| O_3_ | 6.917 | 0.917 |
| O_4_ | 6.938 | 0.938 |

Note: the numbering scheme of O atoms is as shown **Figure 3f**. Although Bader charges are grid-based partitions that generally underestimate formal ionic valences due to electron sharing and basis set overlaps, the observed electron redistribution supports the assignment of Cu in an oxidized state, consistent with the electron depletion inferred from our XPS measurements.

**Table S3.** Performance of the formaldehyde sensors shown in **Figure 4b**.

| **Material** | **Response** | **Concentration (ppm)** | **Operation Temp. (°C)** | **Ref. No.** |
| --- | --- | --- | --- | --- |
| Au single-atoms@In_2_O_3_ nanosheets | 10 | 5 | 100 | 25 |
| Au-loaded ZnO nanorod arrays | 6 | 1 | 70 | 70 |
| Spherical SnO_2_@graphene | 4.9 | 1 | 120 | 71 |
| Co-doped In_2_O_3_ Nanorods | 3 | 1 | 130 | 72 |
| Graphene oxide/SnO_2_ nanofibers | 3 | 1 | 120 | 73 |
| Au@SnO_2_ core-shell | 2.9 | 50 | 25 | 74 |
| NiO-SnO_2_ nanospheres | 2.5 | 1 | 100 | 75 |
| ZIF-67 | 2.2 | 5 | 150 | 76 |
| 3D ZnO nanorods | 1.63 | 1 | 25 | 77 |
| Hierarchical SnO/SnO_2_ nanoflowers | 10 | 1 | 120 | 78 |
| MnO_2_-SnO_2_ | 11 | 1 | 80 | 79 |
| In_2_O_3_@ATQ-rGO | 2.4 | 1 | 25 | 80 |
| CeO_2_/In_2_O_3_ | 5.78 | 1 | 25 | 81 |
| **Cu_1_-Co_3_O_4_** | **19.1** | **1** | **75** | **Our work** |
| **CuO_NP_-Co_3_O_4_** | **5.40** | **1** | **75** | **Our work** |
| **Pure Co_3_O_4_** | **3.35** | **1** | **75** | **Our work** |

Table S4: Cross-sensitivity of Cu_1_-Co_3_O_4_ toward common interferents at 1 ppm. Values are taken from Figure 4d. The selectivity ratio is defined as Response_Formaldehyde_ divided by Response_Analyte_ at 1 ppm.

| **Analyte** | **Response @ 1 ppm** | **Selectivity vs. formaldehyde** |
| --- | --- | --- |
| Formaldehyde | 6.09 | - |
| Acetone | 0.24 | 25.0 |
| Toluene | 0.03 | 203.0 |
| Ethanol | 0.13 | 46.8 |
| NO | 0.13 | 46.8 |
| Acetaldehyde | 0.52 | 11.7 |
| NH_3_ | 0.10 | 60.9 |
| CH_4_ | 0.04 | 152.3 |
